# Sampling from four geographically divergent young female populations demonstrates forensic geolocation potential in microbiomes

**DOI:** 10.1038/s41598-022-21779-z

**Published:** 2022-11-03

**Authors:** Thomas Clarke, Lauren Brinkac, Chris Greco, Angela T. Alleyne, Patricio Carrasco, Carolina Inostroza, Tiiseto Tau, Wichaya Wisitrasameewong, Manolito G. Torralba, Karen Nelson, Harinder Singh

**Affiliations:** 1 J. Craig Venter Institute, Rockville, MD 20850 USA; 2 Noblis, Reston, VA 20191 USA; 3 Department of Biological & Chemical Sciences, Faculty of Science and Technology, The University of the West Indies, Cave Hill Campus, Bridgetown, Barbados; 4 Faculty of Dentistry, Centro de Investigación en Biología y Regeneración Oral (CIBRO), Universidad de los Andes, Santiago, Chile; 5 Department of Virology, Sefako Makgatho Health Sciences University, Pretoria, South Africa; 6 South Africa Medical Research Council, Pretoria, South Africa; 7 Department of Periodontology, Faculty of Dentistry, Chulalongkorn University, Bangkok, Thailand

**Keywords:** Microbial communities, Microbial ecology

## Abstract

Studies of human microbiomes using new sequencing techniques have increasingly demonstrated that their ecologies are partly determined by the lifestyle and habits of individuals. As such, significant forensic information could be obtained from high throughput sequencing of the human microbiome. This approach, combined with multiple analytical techniques demonstrates that bacterial DNA can be used to uniquely identify an individual and to provide information about their life and behavioral patterns. However, the transformation of these findings into actionable forensic information, including the geolocation of the samples, remains limited by incomplete understanding of the effects of confounding factors and the paucity of diverse sequences. We obtained 16S rRNA sequences of stool and oral microbiomes collected from 206 young and healthy females from four globally diverse populations, in addition to supporting metadata, including dietary and medical information. Analysis of these microbiomes revealed detectable geolocation signals between the populations, even for populations living within the same city. Accounting for other lifestyle variables, such as diet and smoking, lessened but does not remove the geolocation signal.

## Introduction

The human microbiome is comprised of communities of microorganisms, including bacteria, that live on and in the human body and form distinct ecologies. The human microbiome has been observed to differ between individuals from different geographic locations across multiple body sites, such as stool^1–4^, oral^5,6^, and hair and skin^7,8^ samples. However, any robust detection of the signal would also have to account for potential confounding factors^9–11^. Since many societal norms are correlated with geography^12^, the extent that a specific global position drives the differences measured outside of any lifestyle differences is still incompletely understood. For instance, the differences in microbiomes, as described in Yatsunenko et al.^3^, based on cohorts from the United States of America, Malawi, and the Amazonian region of Argentina, could have arisen either from their respective geographic position or their distinct diets, as a similar divergence in diet was previously shown to alter the microbiome in another study^13^. The microbiome differs in more closely geo-located populations, though the variances can be attributed to different lifestyle such as diet and altitude^2,11,14,15^. In these studies, with limited sample size or locations, both the identification and quantification of their respective contributions to the variation in the microbiomes are difficult to comprehend^2,5,16–18^.

The studies have identified taxa with significantly different abundance in various populations due to differences in lifestyles, such as diet and smoking. For example, the oral cavity of individuals whose diets are rich in carbohydrates harbor a greater abundance of cariogenic bacteria *Lactobacillus* spp. and *Streptococcus mutans*^19^ and lower levels of *Proteobacteria* species in smokers compared to non-smokers^20^. In our previous study, we observed *Peptoniphilus* and *Staphylococcus* as differentially abundant when comparing samples of healthy individuals from Maryland, USA and California, USA based on pubic and scalp hair samples respectively^7^. Taxa that distinguish geographic locations are still poorly documented, with only a few locations being tested, and with these, often any taxon identified is possibly a result of additional metadata variables, such as diet^14^ or oral hygiene^21^.

We recently published the FMD database where we obtained the publicly available microbiota data of various body sites across the multiple countries^22^. The database analysis suggests different microbiota composition across countries, but it is difficult to study the confounding variables due to samples collected, process and sequences using different protocols. To overcome these challenges and in addressing the geolocation potential of microbiota in forensics, we obtained oral and stool samples from four different countries across four different continents along with lifestyle metadata including diet and other lifestyle variables. All the participants in this study were healthy females between the ages 18–30 who were born in the location sampled to further remove possible confounding variables of age and gender. We observed there are significantly abundant taxa which can help predict the differences in the taxonomy that are changed by divergences in lifestyles; and identify which of the taxa in the microbiome are important for distinguishing the geographies. We further demonstrate the extent that these datasets can show more fine-scale geospatial resolution by comparing samples from different neighborhoods.

Multiple techniques are available to analyze the communities to document differences between microbiomes harvested from different populations. Total OTU tables can be examined, as with Permutational Multivariate Analysis of Variance (PERMANOVA)^23^, or the sets of taxa can be compared, either using a phylogenetically-dependent distances such as UniFrac^24^ or a phylogenetically-independent metric such as Bray–Curtis^25^. With these tools, it is possible to document the multiple factors such as diseases^26,27^, pet ownership^9,28^, and local environment^29,30^, that can influence the composition of the microbiota that can distinguish populations. This has led to multiple proposals for the capacity of the microbiomes as a forensic tool with which to identify people^31–35^.

## Methods

### Ethics statement

The study was approved by the J. Craig Venter Institute (JCVI) Institutional Review Board (No. 2015-219), University of Los Andes Health Center at San Bernardo Ethics Committee (No. CEC201627), University of the West Indies-Cave Hill/Barbados Ministry of Health Research Ethics Committee (No. 170104-A), Sefako Makgatho University Research Ethics Committee (No. SMUREC/M/91/2017:IR), and the Human Research Ethics Committee of Chulalongkorn University (No. HREC-DCU 2018-090). All methods were performed in accordance with relevant guidelines and regulations. Written informed consent was obtained from all participants prior to sample collection.

### Cohort description and sample collection

Paired buccal mucosa (oral) and stool samples were collected from adult females (18–26 years old) who were born and raised in one of the following regions of the world: Barbados (*n* = 32); Santiago, Chile (*n* = 69); Pretoria, South Africa (*n* = 37); and Bangkok, Thailand (*n* = 68). Participants had no history of major diseases in the past year (i.e., irritable bowel syndrome and inflammatory bowel disease) and were not biologically related. Participants currently taking antibiotics were excluded from the study. A lifestyle behavioral questionnaire was completed by each participant at enrollment. Body mass index (BMI; kg/m^2^) was calculated for each participant and categorized according to the World Health Organization classification scheme: ≥ 30 = obese; 25–29.9 = overweight; 18.5–24.9 = normal; ≤ 18 = underweight (World Health Organization 1995). Samples were self-collected using the OMNIgene® ORAL and OMNIgene® GUT collection kits (DNA Genotek, Ontario, Canada) following protocol without modification. One hour prior to oral specimen collection, participants refrained from eating, drinking, smoking, or chewing (i.e.*,* gum and tobacco). All samples were stored at − 20 °C for up to 7 days, followed by long term storage at − 80 °C without a freeze–thaw cycle until DNA extraction.

### Sample preparation and DNA isolation

Oral and stool samples were thawed on ice prior to DNA extraction. DNA was extracted using the DNeasy Powersoil DNA Extraction kit (Qiagen Inc, Hilden, Germany) to generate high molecular weight DNA free of PCR inhibitors. Samples were examined for DNA integrity by agarose gel electrophoresis and Nanodrop (ThermoFisher Scientific, Waltham MA). DNA was quantified using SYBR Gold (ThermoFisher) prior to downstream applications.

### 16S rRNA gene V4 sequencing

Microbiota profiling was performed targeting the V4 region of the 16S rRNA gene^36^. 16S rRNA gene amplification in each sample was performed using adaptor and barcode ligated V4 specific primers so that sequences from each sample in the library were identified with unique barcode indices. Mock community DNA was included in the library preparation step as described previously in Kozich et al*.*^37^. The mock community serves as a control for contaminants as well as a tool to ensure reproducibility and quality sequence reads, indicating the presence of unexpected spurious operational taxonomic units (OTUs). In addition, PhiX DNA was spiked into all sequencing runs as an integral control for sequencing. A high percentage of PhiX spikes (10–20%) adds diversity to 16S rRNA gene runs and improves quality. Amplicon from extraction controls and no template controls were also included to determine if any contamination occurred during DNA extraction or during the library prep stage. 16S rRNA gene libraries were analyzed on the High Sensitivity DNA Chip (Agilent, Inc. Santa Clara, CA) to ensure that libraries were free of adapter dimers contaminants and that they were appropriately sized for the platform. 16S rRNA gene libraries were sequenced using v2 chemistry 2 × 250 bp format 500 cycles on Illumina MiSEQ (Illumina Inc, La Jolla, CA) using standard manufacturer’s specifications. QC analysis was performed after each sequencing run where the % reads ≥ Q30, passing filter clusters and yield/sample were monitored.

### 16S rRNA gene sequence data analysis

Sequence reads from the 206 samples obtained plus 11 negative controls were processed using an in-house 16S rRNA gene data analysis pipeline. Sequencing from all the samples averaged 15,649 reads before mapping (Table S16). OTUs were generated using the default parameters in UPARSE^38^ and taxonomies were assigned to these OTUs with mothur^39^ using version 123 of the SILVA 16S rRNA gene database^40^ as the reference database. All samples that contained less than 2000 paired reads, with only stool or oral or which had incomplete metadata were removed. Additionally, OTUs with less than ten total reads were removed. This left 197 samples that were further considered for downstream analysis, which were normalized to relative abundances of reads mapping to different taxa at all taxonomic levels using the R-package Phyloseq^23^. Overall, the passing samples averaged 12.094 mapped reads. There were 291 species that had OTUs mapped to both stool and cheek microbiomes (Fig. S1).

### Statistical analyses

The 16S data and the differences between different geographic locations was analyzed using a variety of techniques, including visualization of the principal component analysis (PCA) and permutational multivariate analysis of variance (PERMANOVA). Distances between microbiomes were calculated using the VEGAN R-package using Bray–Curtis dissimilarity matrix^41^. Differentially abundant genera were identified using DESeq2 package version 1.12.3 in R^42^ using a FDR cutoffs as calculated by DESeq2 using the Benjamini and Hochberg False Discovery Rate^43^. MaAsLin2 was used to determine the multivariable association between phenotypes and microbiome abundance (34784344). Principle component analysis and the Pearson’s correlation of the metadata variables were calculated in Python using scikit-learn^44^ and scipy^45^.

## Results

### Cohort demographics

A total of 206 female participants were enrolled in the study and passed our quality control standards. All participants were required to be between the ages of 18–26 years old (22.5 ± 2.1) and to be born and at the time living in one of four geographically distinct regions of the world: Barbados; Santiago, Chile; Pretoria, S. Africa; and Bangkok, Thailand. The regions do, however, differ by an order of magnitude in their geographic spread as the intra-distance separating the residence neighborhood of participants ranged from 34 (Barbados) to 681 km (Pretoria, S. Africa) (Fig. S2). The Chilean and the South African datasets are further divided into two contiguous sub-regions, or neighborhoods, to allow for a micro-geographic analysis. The study population is largely dominated by individuals with self-identified Thai heritage (33%), followed by Black African (16%), Afro-Caribbean (14%) and white (14%) descent, although 19% of the Chilean population did not report ethnicity.

Study participants, despite the divergent geographies, mostly have similar dietary and lifestyle habits (Table S1). Over half the study population (62%) have a normal BMI, with the mean BMI in this range (22.6 ± 5.5). The diets of the different cohorts are also similar as of the total cohort, 78% consume a starch heavy diet (≥ 4 days a week) of rice, bread and pasta, followed by 66% who frequently consume (≥ 4 days a week) vegetables and fruit and 49% who frequently consume dairy products. The study population is split by level of tobacco exposure, with 51% of the population having never smoked, and 43% being exposed to second-hand smoke through living with a smoker. Over half (56%) of the study population own one or more pets.

### Stool microbiome

**Figure 1:**
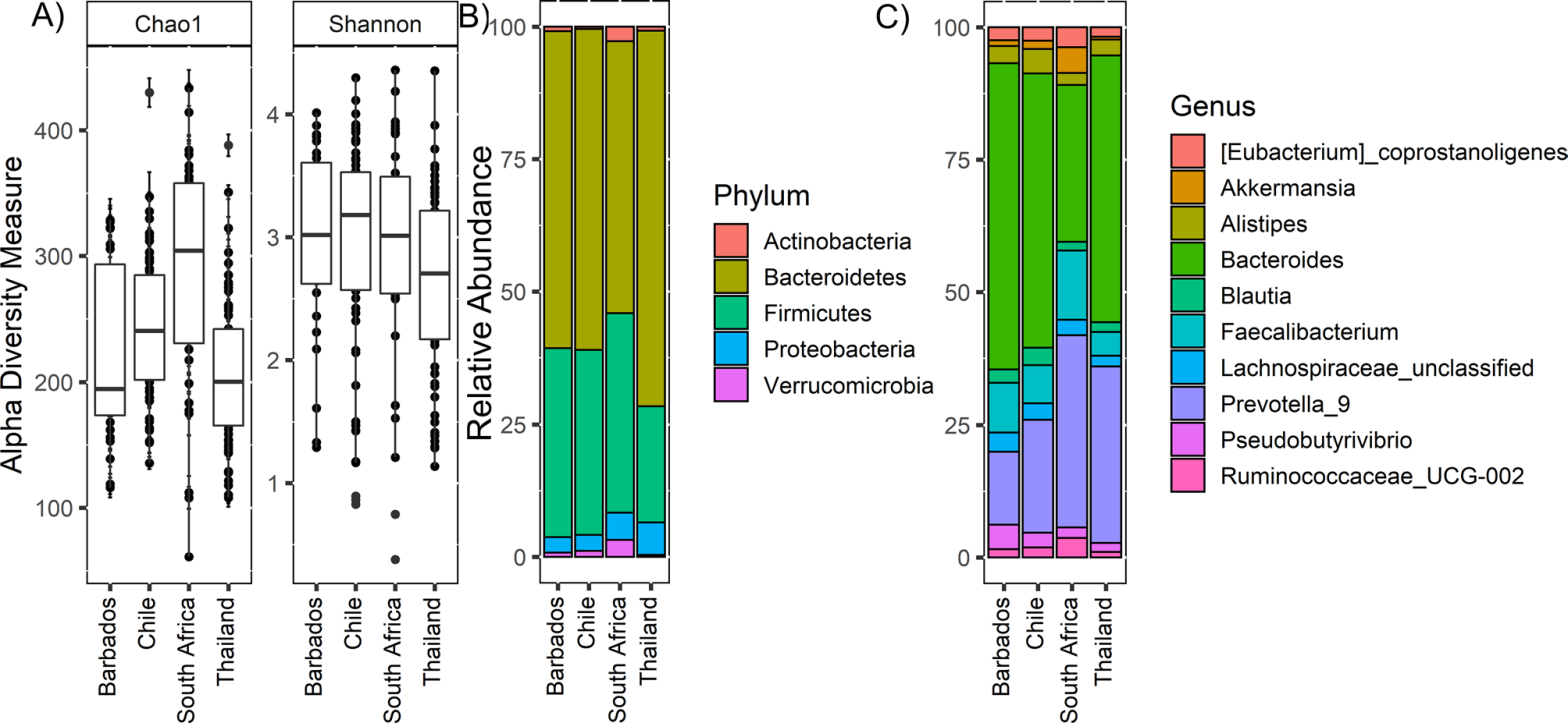
Stool alpha diversity: (**A**) microbial richness and evenness of cheek was calculated based on the Chao1 and Shannon index of four different sites. The y-axis represents the alpha diversity unit scale either Shannon or Chao1. (**B**) Phylum level abundance of stool samples, (**C**) top ten most abundance genera in stool samples.

The OTUs identified using the UPARSE pipeline^17^ were used to compute the alpha diversity of the microbial communities using the Chao1 (species richness) and Shannon (species evenness) indices. The mean Shannon indices reveal that the microbiota diversity is only significant between Thailand-Chile with FDR < 0.05. In case of Chao1 diversity index Thailand-Chile, Thailand-South Africa, Chile-South Africa, Barbados-South Africa have different richness with FDR < 0.05 (Fig. 1A).

**Figure 2:**
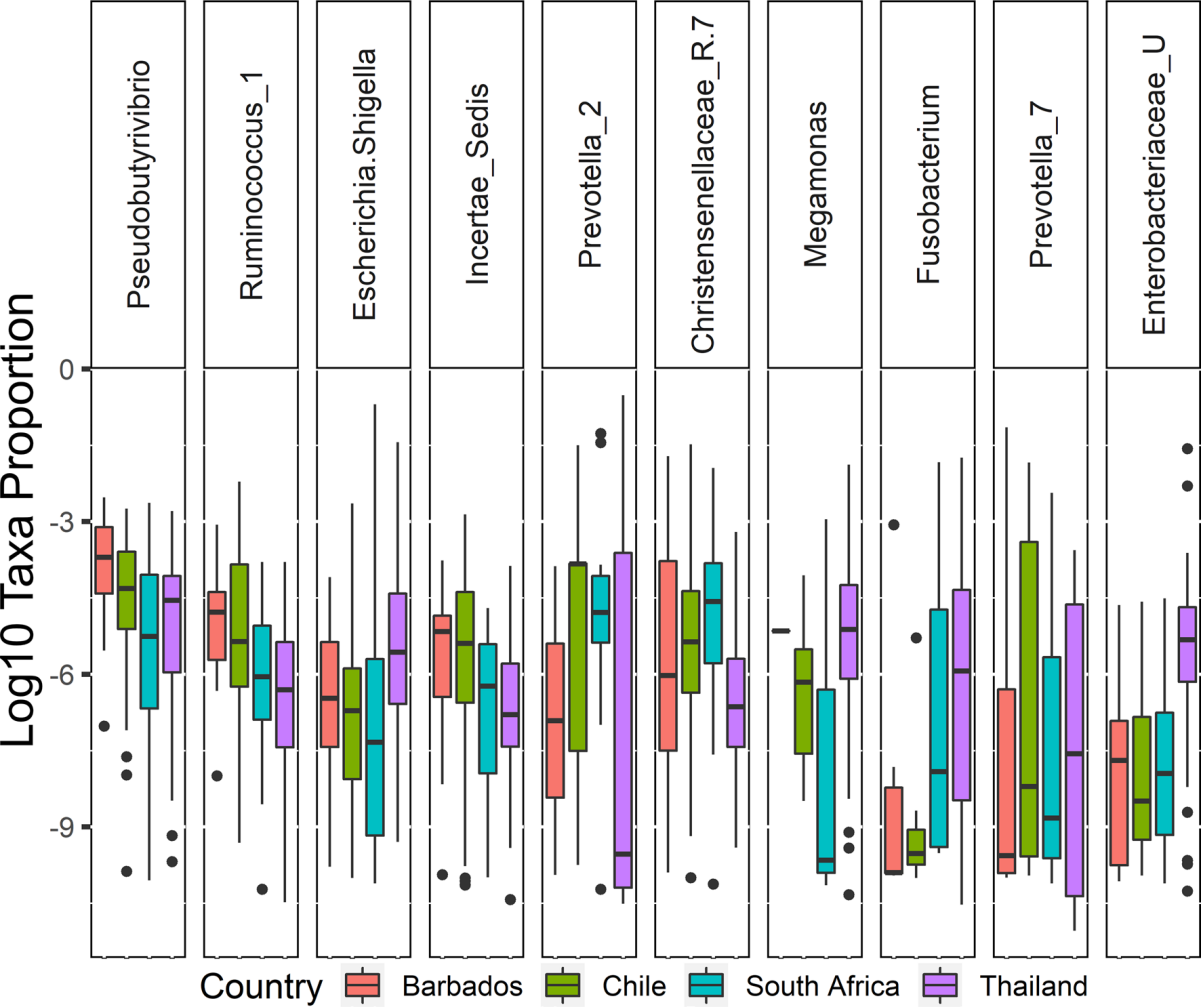
The significant differential abundant stool genera between four countries displayed as Box and whisker plot.

The three abundant phyla (Actinobacteria, Bacteroidetes, Verrucomicrobia) have significant differential abundance with FDR < 0.05 among the four countries (Fig. 1B). The top five most dominant taxa identified among stool microbiota are *Bacteroides*, *Prevotella_9*, *Faecalibacterium*, *Alistipes*, and unclassified *Eubacterium* (Fig. 1C). Interestingly, *Faecalibacterium,* an anti-inflammatory commensal recognized for its importance in maintaining intestinal health (see Miguel et al.^46^), is observed at significantly higher abundance in South African individuals and lower abundance in the Thai individuals (Table S2). There are 28 differentially abundant genera between the four-country using DESeq2 algorithm with only five genera have high abundance in the stool microbiome. These are *Pseudobutyrivibrio, Fusobacterium, Christensenellaceae_R-7_group, Ruminococcus_1, Escherichia-Shigella* and other important ones are *Prevotella, Incertae_Sedis, Megamonas, Enterobacteriaceae_unclassified* (Fig. 2). The data suggest that in these populations with relatively similar diets (Table S1), the most geographically distinct taxa (Table S6) are in lower abundance in the stool representing only 10.4% of the total gut microbiota. Using Pearson’s Correlation calculated between the first five Principal Components (PCs), we examined the influential factors of lifestyle behaviors on the composition of microbial communities originating from stool among the entire study population of Barbadian, Chilean, Pretorian and Thai individuals. The composition of stool microbiota across all the populations is most influenced by BMI (PC4 p = 0.018, r^2^ = 0.029; 3.35% variance). Within single region populations, Chilean stool microbiota correlates with having never smoked (PC3 p = 0.0271, r^2^ = 0.074; 4.02% variance), and Pretorians being the only population with stool microbiota that correlates with BMI categories (PC1 p = 0.0205, r^2^ = 0.156; 67.62% variance) and the frequency of eating corn/cornmeal (PC3, p = 0.0077, r^2^ = 0.196; 4.02% variance). The Thai population’s stool microbiota is correlated with living with a current smoker (PC3 p = 0.012, r^2^ = 0.093; 5.53% variance) and being an ex-smoker (PC4 p = 0.0097, r^2^ = 0.0998; 4.56% variance). Stool microbiota of the Barbadian population is not significantly correlated with any of the lifestyle behavioral factors tested.

### Oral microbiome

**Figure 3:**
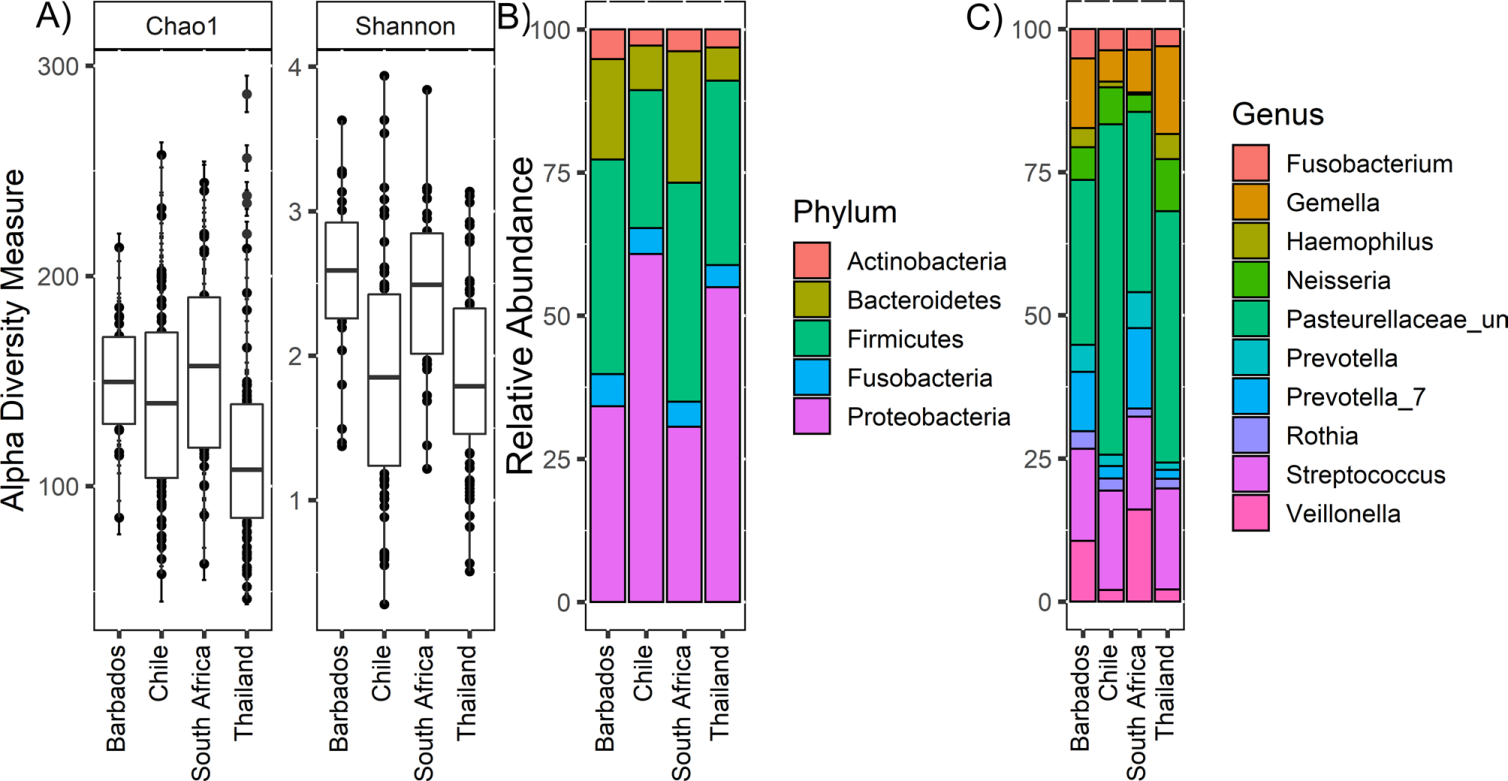
Cheek alpha diversity: (**A**) microbial richness and evenness of cheek was calculated based on the Chao1 and Shannon index of four different sites. The y-axis represents the alpha diversity unit scale either Shannon or Chao1. (**B**) Phylum level abundance of Cheek samples, (**C**) top ten most abundance genera in cheek samples.

The mean Chao1 indices reveal that the microbiota diversity is significant between Thailand–Barbados, Thailand–Chile, Thailand–South Africa and Chile–South Africa with FDR < 0.05. Whereas only significant difference was observed between Thailand and Chile using Shannon diversity index with FDR < 0.05 (Fig. 3A). Two abundant phyla, Bacteroidetes and Proteobacteria have significant differential abundance between countries (FDR < 0.05) (Fig. 3B).

**Figure 4:**
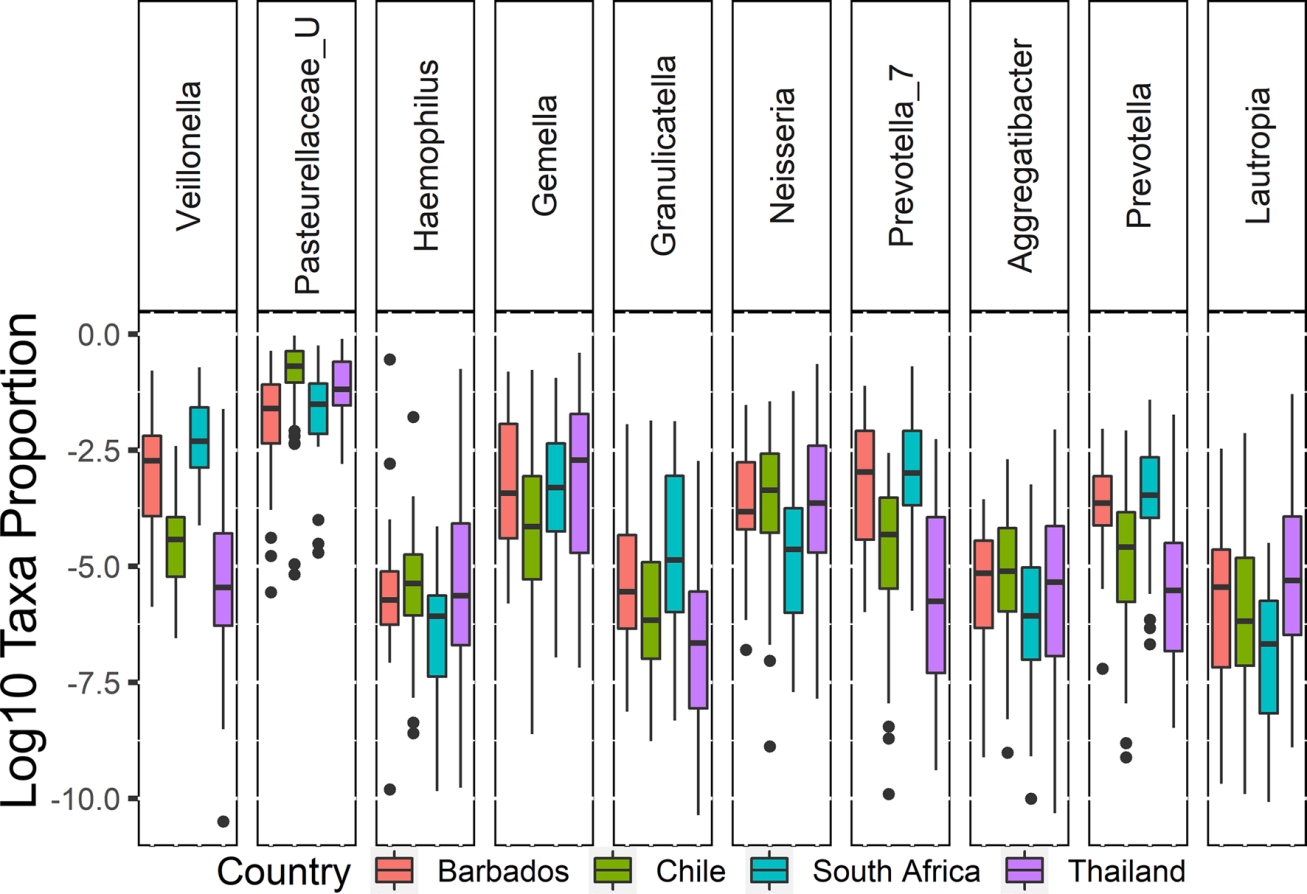
Box and whisker plot showing the significant differential abundant cheek genera between four countries.

The top most dominant taxa identified among oral microbiota are two *Prevotellaceae* genera, *Pasteurellaceae_unclassified*, *Haemophilus*, *Streptococcus*, *Gemelia*, *Veillonella* and *Neisseria* (Fig. 3C), all of which have been documented as among the most abundant in oral microbiota in other populations^47^*.* The oral microbiomes also have thirty-five differentially abundant genera (Table S7). Eight of the ten most dominant genera in the oral microbiota Pasteurellaceae_unclassified, *Streptococcus*, *Gemelia*, *Veillonella,* two *Prevotellaceae* genera, *Haemophilus* and *Neisseria* have significance difference in at least one of the populations with FDR < 0.05 (Fig. 4). As such, the oral microbiome on average contains more bacteria from taxa with geographic specific signals as a percentage of the total microbiome (16%) when compared to percentage of the microbiome in differentially abundant taxa in the stool samples (2%).

We also find that lifestyle and behavior have a greater influence on the oral microbiota compared to stool microbial composition for those factors tested. Like with the stool samples, the oral microbiota composition are associated with different lifestyles and behaviors in different populations, with the exception of BMI which was strongly correlated with oral microbial communities across all four populations using BMI categories: Chile (PC1 p = 0.0085, r^2^ = 0.103; 71.77% variance), S. Africa (PC1 p = 0.0169, r^2^ = 0.242; 37.77% variance,) Barbados (PC1 p = 0.0155, r^2^ = 0.174; 46.41% variance) and Thailand (PC2 p = 0.017, r^2^ = 0.083; 21.83% variance respectively). In addition to BMI, oral microbiota of the Chilean and Thai population correlated with the frequency of consuming fish with p value < 0.05 (PC2 p = 0.033, r^2^ = 0.0710; 14.13% variance and PC3, p = 0.0081, r^2^ = 0.1029; 10.07% variance), while oral microbiota composition of the Barbadian population was also strongly correlated with the frequency of eating meat such as beef and pork (PC2 p = 0.0450, r^2^ = 0.157; 19.01% variance), as well as eating fruits and vegetables (PC4 p = 0.00169, r^2^ = 0.342; 8.97% variance).

### Global geographical variability of oral and stool microbiota

**Figure 5:**
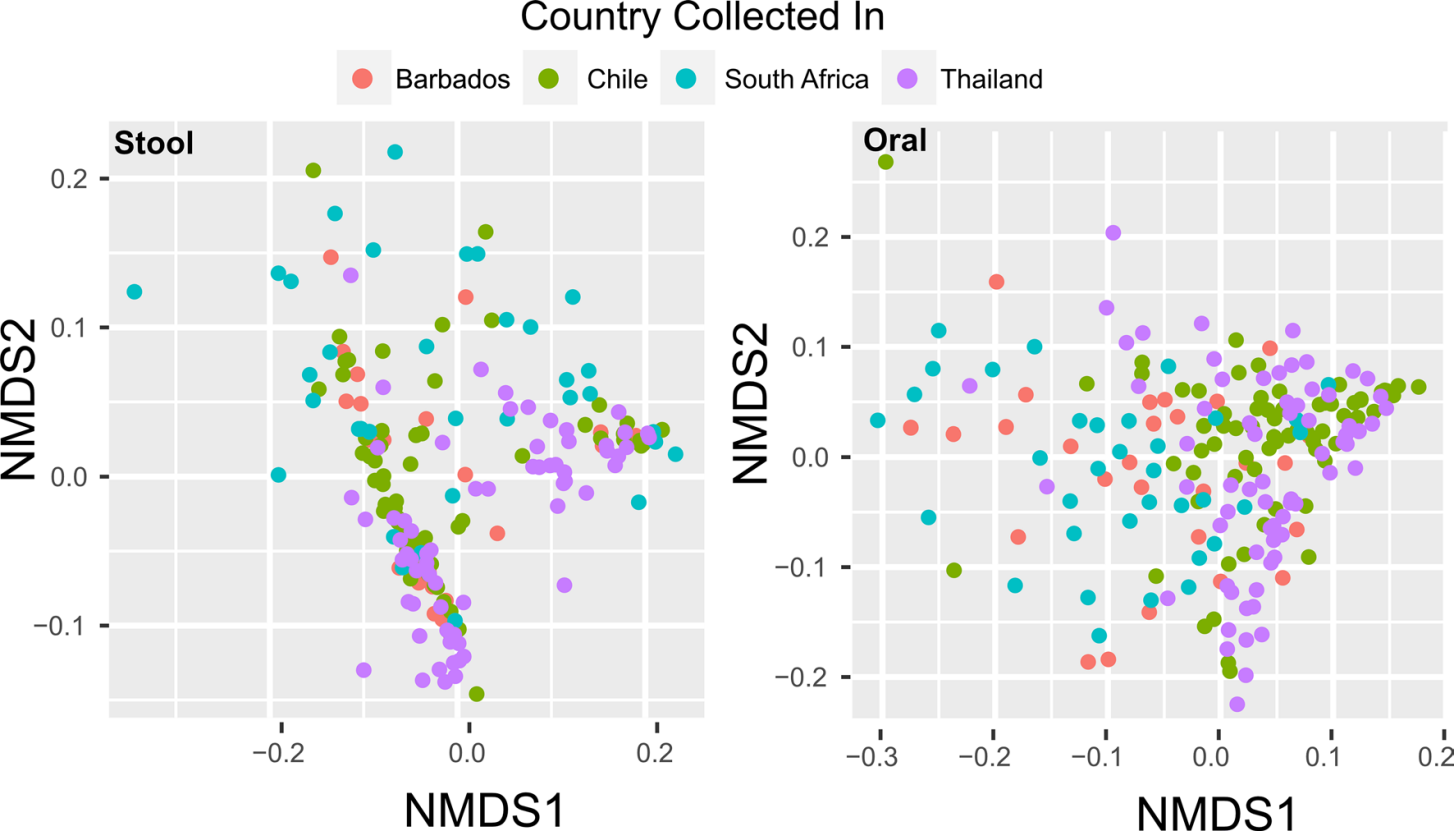
Oral (*n* = 195) and stool (*n* = 196) microbiota differences according to body site and geographical location (Barbados, Chile, Thailand and S. Africa). Measured by NMDS using weighted UniFrac distance in stool (PERMANOVA r^2^ = 0.084, p = 0.001), and oral (PERMANOVA r^2^ = 0.161, p = 0.001).

Both oral and stool microbial communities at genus level exhibited distinct geographic variation (i.e., country of origin) in their taxonomic distribution, though the body site from which the microbial community originated was more discriminatory (Fig. 5). We also identified potential differentially abundant species among the four countries using the usearch “unoise” algorithm to obtain ASVs. Due to skeptical nature of species prediction using short tags V4 regions, the details are described in the Supplementary Tables S8–S15.

Microbiota from the oral cavity can differentiate geographic locations as shown by both NMDS (Fig. 5) and by PERMANOVA, with approximately 16% of the variation between oral microbial communities explained by country of origin. Within the study populations, Chilean oral microbial communities were the most distinct geographically, explaining 17% of the taxa variation, as compared to 9% for Pretorian and 4% for Barbadian oral microbiota. Using only the differential abundant taxa in the oral microbiome, the country of origin is less explanatory explaining only 11% of the variation by PERMANOVA. Country of origin explained less than 8% of that variance in the taxonomic distribution of the stool, with insufficient differentially abundant taxa to run PERMANOVA on this reduced set.

Since it is possible that the differences could derive not from differences in geographic locations, but instead differences between the lifestyles of the cohorts, we also examined the effect of the metadata values on the strength of the PERMANOVA signal. For all of the metadata variables in the oral and stool microbiome, a significant signal differentiating the country by PERMANOVA remains even after accounting for the metadata (Table S5). The strength of this signal is not similarly observed using only the metadata or the combined data, suggesting that the geographic signal is strongest. However, in metadata variables previously found to be influential in sculpting the microbiome, such as smoking for the oral microbiome^20,48^, and BMI for the gut microbiome^49,50^, the PERMANOVA signal remains strong. Interestingly, the strongest reduction of the signalin the oral microbiome and a significant reduction in the stool microbiome is in connection with how much beef or pork an individual eats per week. Previous work on the effect of a carnivorous diet on the oral microbiome was inconclusive^6,51,52^, though these have mostly concentrated on vegan versus omnivore diets.

We also investigated if the geolocation signal could be amplified either by using differentially abundant taxa or by combining multiple body sites. When only taxa identified as differentially abundant in at least one location compared to the other locations were used, there was an increase in the PERMANOVA signal in both the stool (25%) and oral (54%) microbiome (Fig. S3). However, combining the taxa distribution of oral and stool samples across geography either by adding the distances or by concatenating the taxa counts, when possible, does not increase the geolocation significance of the combined sample (Table S4). Instead, each of the combined sample averages out to below the significance of the oral signal, suggesting that oral microbiota alone has higher geolocation prediction power as compared to stool and combined body sites.

### Intra-region geospatial variation of oral and stool microbiota

**Figure 6:**
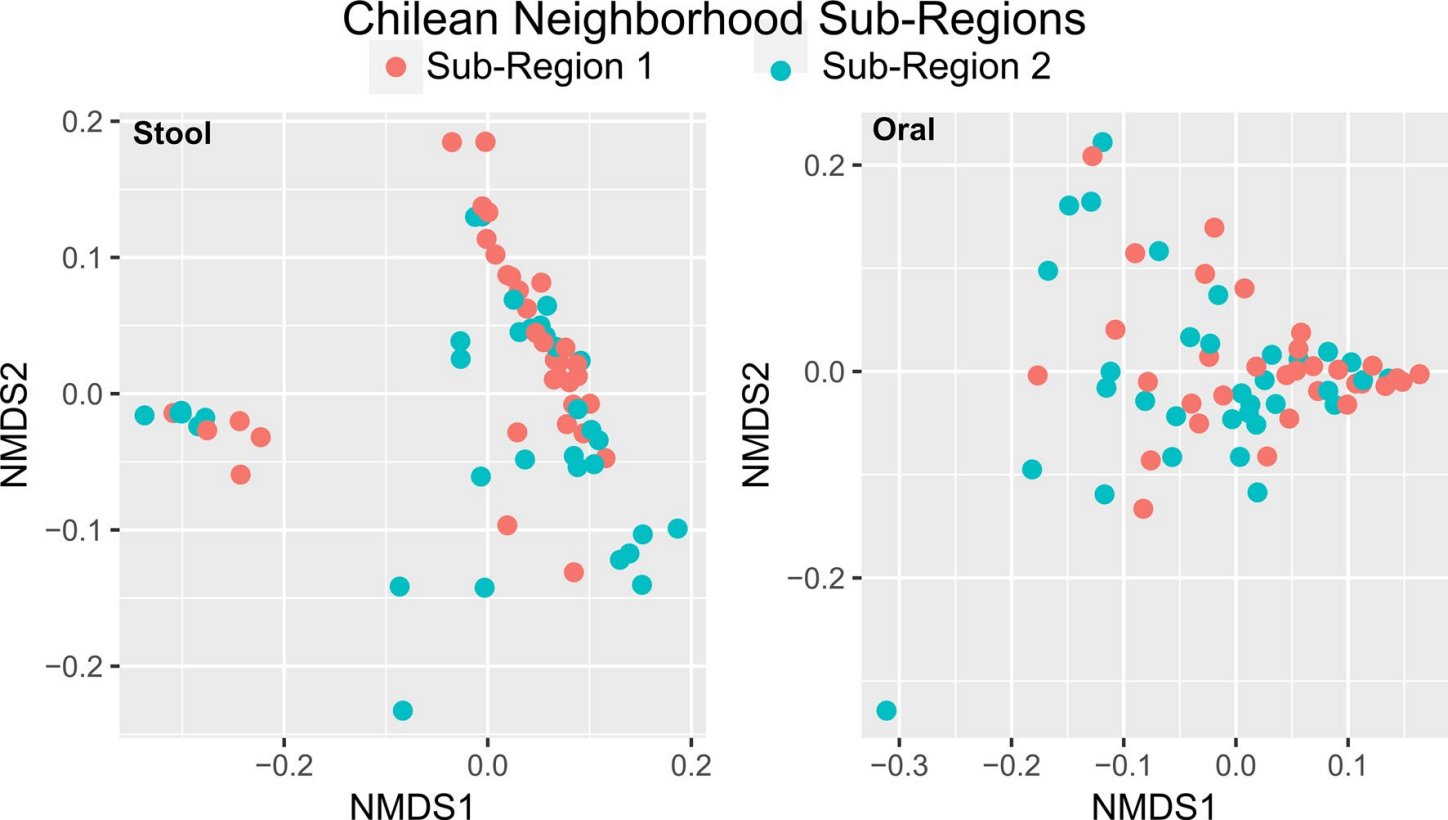
Oral (*n* = 66) and stool (*n* = 67) microbiota diversity between populations from different neighborhoods (sub-region 1 and sub-region 2) in Santiago, Chile as shown by NMDS using weighted UniFrac distance (stool: PERMANOVA r^2^ = 0.026, p = 0.159; oral: PERMANOVA r^2^ = 0.032 p = 0.089). The boundaries of the neighborhoods are shown in Supplementary Fig. S2B.

To assess the extent of variation of oral and stool microbial communities within a geographical region, Chilean and Barbadian study populations were each divided into two distinct neighborhood sub-regions ranging from 27.5 to 178 km based on their residence (Fig. S2). Neighborhood sub-regions were determined by prioritizing geographically discrete and continuous sub-regions with near equal subject populations, without considering any metadata and sociological differences. The Chilean neighborhoods do not have a significant difference between oral or stool microbiomes as identified by PERMANOVA (Fig. 6). Only one of the taxa (*Family XI Gemella)* was one of the top five taxa in the Chilean oral microbiome (Fig. 3), and differentially abundant between the two sub-regions. Though the two from the stool microbiomes were less abundant. There were no taxa that globally differentially abundant. The microbial communities of the Barbadian population had an overall similar level of difference between the neighborhood sub-regions as did the Chilean population even with a smaller geographical range (27.5 to 32.6 km), though the lower number of subjects does limit the significance of these differences (Fig. S4). No taxa in the Barbados oral samples were identified as significantly differentially abundant, with the exception of one stool taxa (*Prevotellaceae Prevotella v9*) (Fig. 1). This taxa is associated with carbohydrate-rich diets^53^.

However, similar to previous studies of populations living in the same country^2,14^, when considering the lifestyle behaviors of the individuals resident in each sub-region, some significant differences emerge. Sub-region 1 and 2 in Santiago Chile have different economic resources as is reflected in their cultural and dietary choices^54^, in addition to the microbiomes. For example, residents in Chilean sub-region 1 more frequently consume fruits/veggies (p = 0.0027) and have a lower BMI (p = 0.001) than those resident in Chilean sub-region 2, while there are more pet owners in sub-region 2 than in sub-region 1 (p = 0.0098). Within the Barbadian population, residents in neighborhood sub-region 1 more frequently consume fish (p = 0.0014) and have a higher BMI (p = 0.0124). When accounting for the metadata differences, the size of the geolocation effect did not appreciably decrease for any of the sub-region comparisons nor any of the body sites. Likewise, the effect size on the microbiome based on the differences in metadata for the groups usually was small, with r^2^ almost always around 0.01, with the singular exception of the weekly consumption of bread, rice, and pasta between the two populations in the two sub-regions in Barbados (r^2^ = 0.097). It would be interesting to know what this dietary difference can further be attributed to food costs, and whether it can be used in the process of forensic identification of the victim similar to as in Chile.

## Discussion

As more and more varied microbiome datasets are made publicly available, the potential to use these datasets in conjunction with evidence as a forensic tool similarly increases, as shown in Cho et al.^55^. However, the usefulness of this data is dependent also on the development of tools to successfully mine the required forensic information from the evidence reproducibly and at a statistically negligent error rate. The dataset presented here could be a valuable resource for the development of such tools, especially for identifying geolocation signals though in the absence of these tools, the data should be used cautiously. The implementation of such a tool requires the combination of the populations, representing a wide range of locationsm while also maintaining a constancy of sequencing and data analysis, such as keeping gender and age range consistent. Likewise, the metadata collected provide detailed information of the potential confounding variables that need to be accounted for when examining the relationship between the microbiomes and the originating geography. Finally, the sequencing of multiple body sites can allow for a comparison of both comparative strength of either and the extent that these signals can be combined to obtain a stronger signal.

Our initial examination of the datasets presented here demonstrates both the presence of a geolocation signal that is independent of population metadata, such as diet and smoking, though these can decrease the signal. This signal is further amplified when the dataset is reduced to the differentially abundant taxa. Microbiomes have previously been shown to separate non-human environments such as offices and dorm rooms^56,57^, suggestive of geographically-specific bacteria. It is possible that these are integrated into human microbiomes, though which ones and to what extent which of these taxa are similarly geo-predictive in different locations across the world remains unanswered. It is possible that combining the analysis presented here with other diverse geolocated microbiomes in a meta-analyses such as Cho et al.^55^ could further elucidate geo-predictive taxa. This would require careful combination of the microbiome results and the metadata from the different studies, while correcting as best possible for the confounding variable, all of which is beyond the scope of this manuscript. Machine learning algorithms, such as random forest, are becoming increasingly common in detecting and decoding signals within microbiomes (reviewed in Ref.^58^). While we did not explore the extent these could successfully separate the locations nor how algorithms trained on this data succeed from additional microbiomes, we suggest that our results support the data as a valuable resource for the future development and testing of a robust algorithm for detection of geographic signatures in human microbiota.

## Supplementary Information

Supplementary Information.

## Data Availability

Raw datasets and associated metadata generated and analyzed as part of this study are available in the NCBI SRA database under NCBI Bioproject PRJNA545251. Processed datasets can be analyzed in comparison with other publicly available human microbiota data through the Forensic Microbiome Database (FMD) located at http://fmd.jcvi.org/. The samples numbers used in the study are described in Supplementary Table S16.
