# Author Correction: Sampling from four geographically divergent young female populations demonstrates forensic geolocation potential in microbiomes

**DOI:** 10.1038/s41598-022-26505-3

**Published:** 2022-12-20

**Authors:** Thomas Clarke, Lauren Brinkac, Chris Greco, Angela T. Alleyne, Patricio Carrasco, Carolina Inostroza, Tiiseto Tau, Wichaya Wisitrasameewong, Manolito G. Torralba, Karen Nelson, Harinder Singh

**Affiliations:** 1 J. Craig Venter Institute, Rockville, MD 20850 USA; 2 Noblis, Reston, VA 20191 USA; 3 Department of Biological & Chemical Sciences, Faculty of Science and Technology, The University of the West Indies, Cave Hill Campus, Bridgetown, Barbados; 4 Faculty of Dentistry, Centro de Investigación en Biología y Regeneración Oral (CIBRO), Universidad de los Andes, Santiago, Chile; 5 Department of Virology, Sefako Makgatho Health Sciences University, Pretoria, South Africa; 6 South Africa Medical Research Council, Pretoria, South Africa; 7 Department of Periodontology, Faculty of Dentistry, Chulalongkorn University, Bangkok, Thailand

Correction to: *Scientific Reports* https://doi.org/10.1038/s41598-022-21779-z, published online 03 November 2022

The original version of this Article contained an error in the spelling of the author Carolina Inostroza which was incorrectly given as Carolina Inotroza.

The original Article has been corrected.

