# Supplementary material for: Sampling from four geographically divergent young female populations demonstrates forensic geolocation potential in microbiomes
Source: Sci Rep. 2022 Nov 3;12:18547. doi: 10.1038/s41598-022-21779-z (PMC9633824; doi:10.1038/s41598-022-21779-z)
Supplement: Supplementary file 1 — Supplementary Information. [file 41598_2022_21779_MOESM1_ESM.pdf]

Sampling from Four Geographically Divergent Young Female Populations Demonstrates Forensic Geolocation Potential in Microbiomes

Authors
Thomas Clarke[1], Lauren Brinkac[1,2], Chris Greco[1], Angela T Alleyne[3], Patricio Carrasco[4], Carolina Inotroza[4], Tiiseto Tau[5,6], Wichaya Wisitrasameewong[7], Manolito G. Torralba[1], Karen Nelson[1], and Harinder Singh[1]

Supplementary Materials

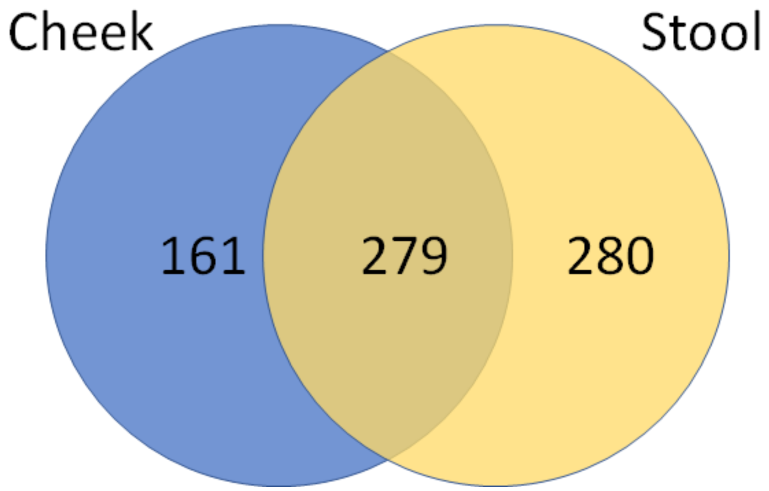

Figure S1. Venn Diagram of Species Counts in the Microbiomes. The number of OTUs assigned to species level taxa to only the stool and cheek FMD microbiomes, and the number shared between the microbiomes.

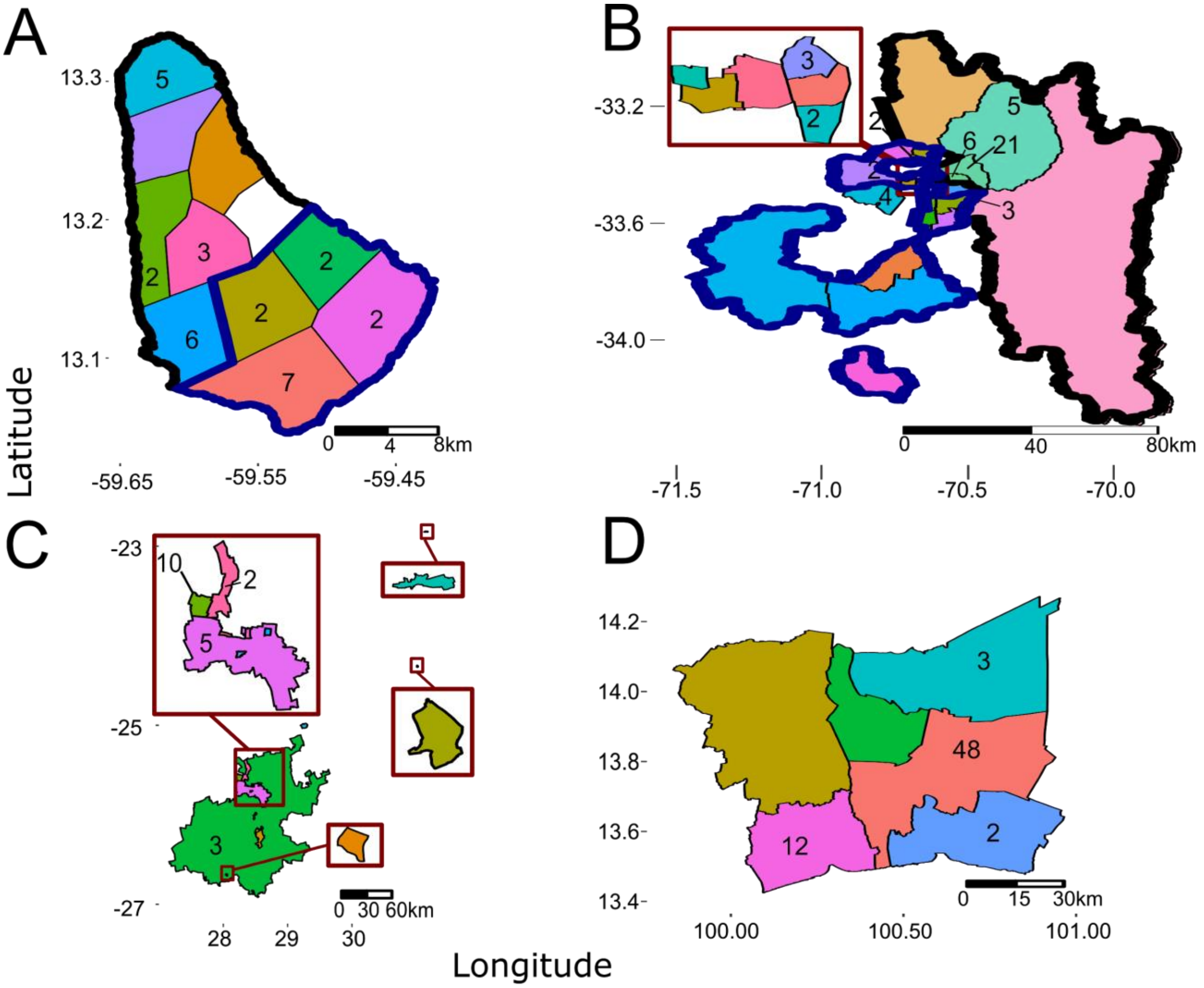

Figure S2. Geographical distribution of the study population. Number of enrolled participants within each residence neighborhood of the following geographical regions: Barbados **(A)**; Santiago, Chile **(B)**; Pretoria, S. Africa **(C)**; and Bangkok, Thailand **(D)**. Distinct sub-regions in Santiago and Barbados used in the intra-city and intra-island comparisons are shown with the thicker width boundaries in black (Region 1) and dark blue (Region 2). Unlabeled colored neighborhoods represent single participant locations. Not shown are three unknown neighborhoods with participants in each of Barbados (*n* = 1), S. Africa (*n* = 2), and Thailand (n = 1).

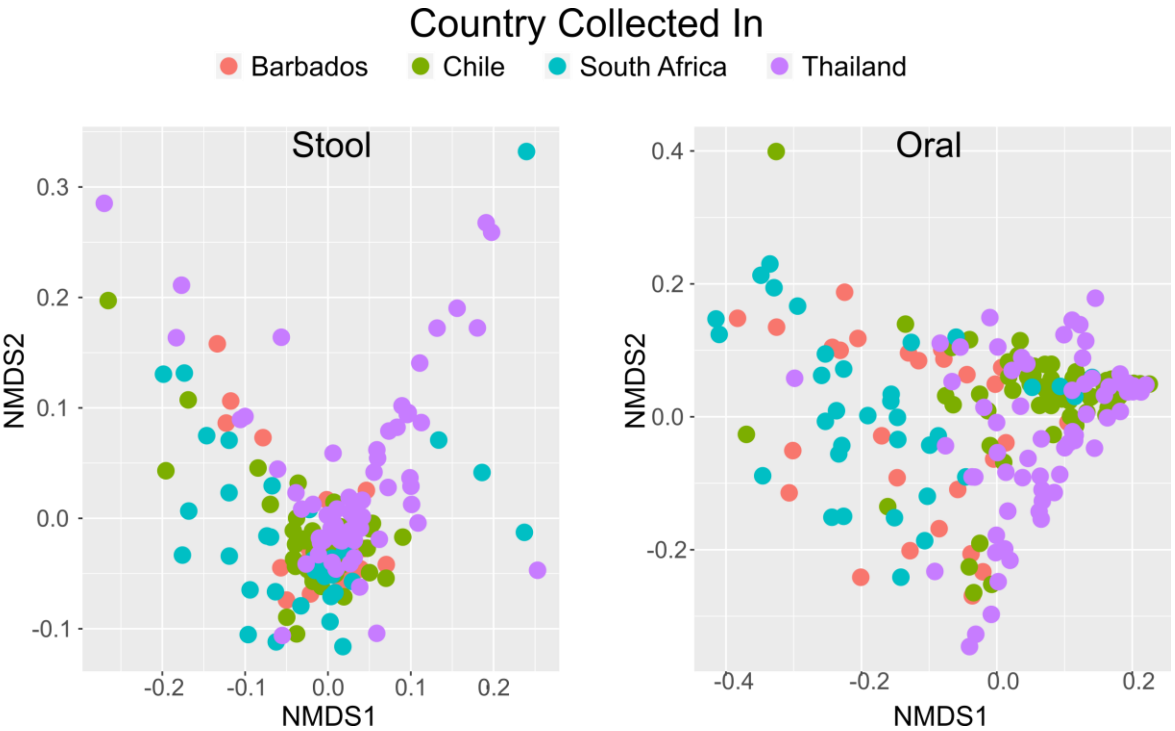

Figure S3. Oral and stool microbiota differences using differentially abundant taxa. Oral (*n* = 195) and stool (*n* = 196) microbiota diversity using only taxa identified as differentially abundant as shown by NMDS using weighted UniFrac distance in stool (# of taxa = 90, PERMANOVA r^2 = 0.105, p = 0.001) (**A**) and oral (# of taxa = 61; PERMANOVA r^2 = 0.248, p = 0.001) (**B**), plots. Differentially abundant taxa were identified by comparisons between each location versus all the others using DESeq2 with a cutoff of FDR corrected p-value of 0.01.

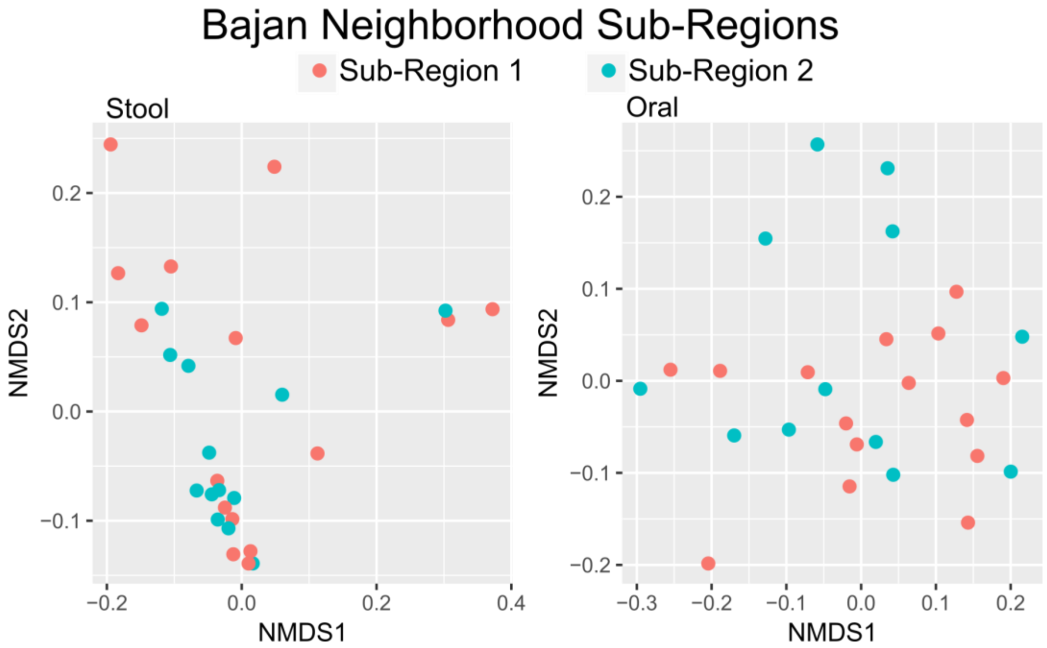

Figure S4. Oral and stool microbiota differences based on neighborhood sub-region located in Barbados. Oral ($n = 26$) and stool ($n = 27$) microbiota diversity between neighborhood sub-regions in Barbados as shown by NMDS using weighted UniFrac distance (stool: PERMANOVA $r^2 = 0.033$, $p = 0.43$; oral: PERMANOVA $r^2 = 0.042$ $p = 0.33$). The boundaries of the neighborhoods are shown in **Supplementary Figure 2A**.

Table S1. Lifestyle behavioral characteristics of the study population

		Geographical location			
		Barbados	Chile	S. Africa	Thailand
No. of participants	[*n* = 206 (100%)]	32	69	37	68
BMI (kg/m2)					
underweight	[*n* = 20 (10%)]	2 (6%)	1 (1%)	1 (3%)	16 (24%)
normal	[*n* = 128 (62%)]	13 (41%)	53 (77%)	20 (54%)	42 (62%)
overweight	[*n* = 30 (15%)]	7 (22%)	10 (14%)	10 (27%)	3 (4%)
obese	[*n* = 10 (5%)]	4 (13%)	1 (1%)	4 (11%)	1 (1%)
Frequency of eating beef/pork					
>7 times per week	[*n* = 32 (16%)]	4 (13%)	10 (14%)	10 (27%)	8 (12%)
4-7 times per week	[*n* = 43 (21%)]	8 (25%)	16 (23%)	3 (8%)	16 (24%)
1-3 times per week	[*n* = 67 (33%)]	11 (34%)	22 (32%)	13 (35%)	21 (31%)
1-3 times per month	[*n* = 47 (23%)]	6 (19%)	15 (22%)	7 (19%)	19 (28%)
never	[*n* = 15 (7%)]	3 (9%)	4 (6%)	4 (11%)	4 (6%)
Frequency of eating poultry					
>7 times per week	[*n* = 27 (13%)]	4 (13%)	3 (4%)	8 (22%)	12 (18%)
4-7 times per week	[*n* = 61 (30%)]	7 (22%)	19 (28%)	9 (24%)	26 (38%)
1-3 times per week	[*n* = 95 (46%)]	19 (59%)	36 (52%)	12 (32%)	28 (41%)
1-3 times per month	[*n* = 14 (7%)]	0 (0%)	6 (9%)	7 (19%)	1 (1%)
never	[*n* = 7 (3%)]	2 (6%)	3 (4%)	1 (3%)	1 (1%)
Frequency of eating fish					
>7 times per week	[*n* = 2 (1%)]	1 (3%)	0 (0%)	1 (3%)	0 (0%)
4-7 times per week	[*n* = 16 (8%)]	1 (3%)	7 (10%)	3 (8%)	5 (7%)
1-3 times per week	[*n* = 91 (44%)]	18 (56%)	32 (46%)	16 (43%)	25 (37%)
1-3 times per month	[*n* = 82 (40%)]	10 (31%)	24 (35%)	15 (41%)	33 (49%)
never	[*n* = 12 (6%)]	2 (6%)	4 (6%)	2 (5%)	4 (6%)
Frequency of eating fruit/vegetables					
>7 times per week	[*n* = 60 (29%)]	19 (59%)	16 (23%)	10 (27%)	15 (22%)
4-7 times per week	[*n* = 75 (36%)]	7 (22%)	27 (39%)	16 (43%)	25 (37%)
1-3 times per week	[*n* = 64 (31%)]	15 (47%)	17 (25%)	9 (24%)	23 (34%)
1-3 times per month	[*n* = 9 (4%)]	0 (0%)	2 (3%)	2 (5%)	5 (7%)
never	[*n* = 0 (0%)]	0 (0%)	0 (0%)	0 (0%)	0 (0%)
Frequency of eating dairy					
>7 times per week	[*n* = 43 (21%)]	9 (28%)	20 (29%)	8 (22%)	6 (9%)
4-7 times per week	[*n* = 57 (28%)]	6 (19%)	11 (16%)	14 (38%)	26 (38%)
1-3 times per week	[*n* = 81 (39%)]	12 (38%)	30 (43%)	13 (35%)	26 (38%)
1-3 times per month	[*n* = 18 (9%)]	4 (13%)	5 (7%)	2 (5%)	7 (10%)
never	[*n* = 5 (2%)]	1 (3%)	2 (3%)	0 (0%)	2 (3%)
Frequency of eating rice, bread, or pasta					
>7 times per week	[*n* = 94 (46%)]	14 (44%)	34 (49%)	20 (54%)	26 (38%)
4-7 times per week	[*n* = 67 (33%)]	11 (34%)	17 (25%)	9 (24%)	30 (44%)
1-3 times per week	[*n* = 38 (18%)]	7 (22%)	12 (17%)	7 (19%)	12 (18%)
1-3 times per month	[*n* = 4 (2%)]	0 (0%)	3 (4%)	1 (3%)	0 (0%)
never	[*n* = 1 (0%)]	0 (0%)	1 (1%)	0 (0%)	0 (0%)
Frequency of eating maize					
>7 times per week	[*n* = 9 (4%)]	0 (0%)	2 (3%)	1 (3%)	6 (9%)

4-7 times per week	[*n* = 24 (12%)]	6 (19%)	7 (10%)	3 (8%)	8 (12%)
1-3 times per week	[*n* = 70 (34%)]	11 (34%)	22 (32%)	18 (49%)	19 (28%)
1-3 times per month	[*n* = 57 (28%)]	7 (22%)	21 (30%)	6 (16%)	23 (34%)
never	[*n* = 43 (21%)]	7 (22%)	15 (22%)	9 (24%)	12 (18%)
Tobacco exposure					
current smoker	[*n* = 17 (8%)]	1 (3%)	8 (12%)	2 (5%)	6 (9%)
ex-smoker	[*n* = 13 (6%)]	2 (6%)	3 (4%)	4 (11%)	4 (6%)
never smoked	[*n* = 105 (51%)]	22 (69%)	32 (46%)	20 (54%)	31 (46%)
live with a current smoker	[*n* = 88 (43%)]	8 (25%)	46 (67%)	9 (24%)	25 (37%)
Pet ownership	[*n* = 116 (56%)]	18 (56%)	53 (77%)	10 (27%)	35 (51%)

Table S2. Relative abundance (%) of top five taxa among Barbadian, Chilean, South African, and Thai stool microbiota

Family, Genus	Barbados	Chile	S. Africa	Thailand
Bacteroidaceae, Bacteroides	41.3	36.8	18.6	38.2
Prevotellaceae, Prevotella_9	9.7	15.0	22.4	24.1
Ruminococcaceae, Faecalibacterium	6.6	5.0	**8.1**	**3.3**
Rikenellaceae, Alistipes	2.3	3.2	1.4	2.3
Ruminococcaceae [Eubacterium]	1.7	1.8	**2.3**	1.4

Significant differentially abundant genera by DESeq2 at p=0.01 between the countries in bold (n = Barbados: 16, Chile: 18, S. Africa: 42, Thailand: 58)

Table S3. Relative abundance (%) of top five taxa among Barbadian, Chilean, South African, and Thai oral microbiota

Family, Genus	Barbados	Chile	S. Africa	Thailand
Pasteurellaceae, unclassified	**16.2**	**45.2**	16.7	33.4
Streptococcaceae, Streptococcus	13.2	15.2	13.4	15.3
Pasteurellaceae Haemophilus	10.4	7.0	9.8	9.5
Family_XI Gemella	9.9	**4.8**	6.1	**13.3**
Neisseriaceae Neisseria	4.9	5.8	2.7	**8.2**

Significant differentially abundant genera by DESeq2 between the countries at p=0.01 in bold (n = Barbados: 8, Chile: 20, S. Africa: 24, Thailand: 51)

Table S4. Geolocation potential (PERMANOVA, r^2) of oral and stool microbiota compared to a combined approach (all values significant p=0.001).

Distance metric	Body site		Combined	
	Stool	Oral	Additive*	Concatenation**
**Bray-Curtis**	0.074	0.150	0.093	0.093
**UniFRAC**	0.099	0.074	0.078	NA
**W-UniFRAC**	0.084	0.161	0.106	NA

*****distance calculated by adding distances; ** distance calculated after combining normalized OTU tables; NA: not applicable

Table S5. Geolocation Potential (PERMANOVA, r^2) for the stool and oral microbiome after accounting for metadata values. Significant values (p.value < 0.05) in the country column indicate the metadata value does not by itself remove the geolocation signal. The country column (PERMANOVA, r^2) consider only country, metadata consider only the respective metadata and country by metadata consider both country and the metadata for calculation of r^2.

	Oral			Stool		
**Metadata Variable**	**Country**	**Metadata**	**Country by Metadata**	**Country**	**Metadata**	**Country by Metadata**
BMI*	**0.161**	**0.011**	**0.033**	**0.084**	0.008	0.016
Never Smoked	**0.156**	**0.014**	0.011	**0.082**	0.006	0.007
Eating Beef/Pork**	**0.115**	**0.052**	0.011	**0.072**	**0.017**	0.016
Eating Chicken/Poultry**	**0.159**	0.012	0.006	**0.078**	**0.021**	0.008
Eating Fish**	**0.154**	0.011	0.009	**0.066**	0.009	0.014
Eating Fruit Vegetables**	**0.150**	**0.016**	0.007	**0.077**	**0.013**	0.011
Eating Dairy**	**0.140**	**0.039**	0.015	**0.085**	0.001	0.014
Eating Rice,Bread,or Pasta**	**0.141**	**0.030**	0.014	**0.069**	**0.018**	0.007
Eating Corn or Cornmeal**	**0.154**	**0.014**	**0.027**	**0.083**	0.005	0.011
Pet Ownership	**0.160**	0.005	0.015	**0.081**	0.004	0.017

bold indicates significance at p = 0.05; * raw values used; ** ranked list used

Table S6. The differentially abundant genera in stool samples obtained using DESeq2 algorithm with FDR < 0.05. The samples are sorted based on baseMean.

Genus	baseMean	log2FoldChange	lfcSE	stat	pvalue	padj
Pseudobutyrivibrio	386.58	-1.32	0.38	-3.46	0.00	0.01
Fusobacterium	163.69	10.04	1.01	9.94	0.00	0.00
Christensenellaceae_R-7_group	159.73	-2.20	0.62	-3.58	0.00	0.01
Ruminococcus_1	141.17	-1.67	0.53	-3.13	0.00	0.01
Escherichia-Shigella	137.67	2.41	0.55	4.36	0.00	0.00
Megamonas	87.57	5.41	1.61	3.37	0.00	0.01
Incertae_Sedis	82.01	-1.38	0.46	-3.03	0.00	0.02
Enterobacteriaceae_unclassified	80.30	2.93	0.71	4.15	0.00	0.00
Prevotella_7	69.75	-12.04	1.33	-9.09	0.00	0.00
Prevotella_2	68.59	11.74	1.95	6.02	0.00	0.00
uncultured	47.56	7.09	2.41	2.94	0.00	0.02
uncultured_bacterium_unclassified	20.85	6.44	1.90	3.39	0.00	0.01
Lachnospiraceae_ND3007_group	20.07	-1.24	0.43	-2.86	0.00	0.03
Holdemanella	19.91	3.43	1.01	3.38	0.00	0.01
Clostridium_sensu_stricto_1	17.20	2.19	0.62	3.53	0.00	0.01
Flavonifractor	17.05	1.23	0.46	2.71	0.01	0.04
Rikenellaceae_RC9_gut_group	12.40	6.67	2.10	3.17	0.00	0.01
Romboutsia	11.06	1.44	0.47	3.08	0.00	0.02
uncultured_organism	10.91	-5.37	1.61	-3.33	0.00	0.01
Butyrivibrio	9.11	-16.58	2.33	-7.13	0.00	0.00

Family_XIII_AD3011_group	8.69	-1.54	0.45	-3.44	0.00	0.01
Bacteroidales_S24-7_group_unclassified	5.49	18.15	2.36	7.71	0.00	0.00
Prevotellaceae_NK3B31_group	3.49	-19.68	2.34	-8.41	0.00	0.00
uncultured	2.44	-1.37	0.48	-2.86	0.00	0.03
Anaerovibrio	2.36	15.30	4.59	3.33	0.00	0.01
Lactobacillus	2.15	3.01	1.08	2.79	0.01	0.03
Marvinbryantia	1.55	-1.53	0.54	-2.85	0.00	0.03
Firmicutes_unclassified	0.67	-2.52	0.75	-3.37	0.00	0.01

Table S7. The differentially abundant genera in cheek samples obtained using DESeq2 algorithm with FDR < 0.05. The samples are sorted based on baseMean.

Genus	baseMean	log2FoldChange	lfcSE	stat	pvalue	padj
Pasteurellaceae_unclassified	13547.33	1.85	0.35	5.32	0.00	0.00
Streptococcus	4510.76	1.43	0.33	4.36	0.00	0.00
Gemella	3308.58	1.33	0.50	2.68	0.01	0.03
Neisseria	1285.63	1.26	0.39	3.26	0.00	0.01
Veillonella	987.80	-1.95	0.35	-5.55	0.00	0.00
Prevotella_7	745.36	-2.22	0.37	-5.98	0.00	0.00
Haemophilus	571.62	1.67	0.50	3.35	0.00	0.00
Prevotella	413.27	-1.27	0.33	-3.90	0.00	0.00
Granulicatella	303.11	-2.16	0.47	-4.64	0.00	0.00
Lautropia	269.56	1.62	0.51	3.20	0.00	0.01
Aggregatibacter	191.55	1.50	0.42	3.59	0.00	0.00
Alloprevotella	174.91	-1.76	0.40	-4.40	0.00	0.00
Bergeyella	137.96	1.56	0.36	4.40	0.00	0.00
Neisseriaceae_unclassified	60.88	0.97	0.39	2.50	0.01	0.05
Selenomonas_3	45.11	-1.48	0.39	-3.80	0.00	0.00
Actinobacillus	43.49	4.27	0.58	7.33	0.00	0.00
Prevotella_6	26.48	-2.68	0.57	-4.69	0.00	0.00
Oribacterium	17.30	-2.50	0.43	-5.79	0.00	0.00
Megasphaera	11.56	-3.28	0.67	-4.87	0.00	0.00
Selenomonas	11.41	-2.24	0.52	-4.29	0.00	0.00
uncultured	9.93	-2.58	0.73	-3.53	0.00	0.00
uncultured	8.86	18.87	2.02	9.32	0.00	0.00
Stomatobaculum	8.45	-1.90	0.48	-3.98	0.00	0.00
Atopobium	8.40	-3.46	0.50	-6.93	0.00	0.00
[Eubacterium]_nodatum_group	8.07	-2.41	0.52	-4.62	0.00	0.00
Dialister	6.35	-2.23	0.47	-4.77	0.00	0.00
Ruminococcaceae_UCG-014	5.88	-1.80	0.45	-4.02	0.00	0.00
uncultured	4.40	4.14	1.19	3.48	0.00	0.00
Catonella	4.06	-1.40	0.42	-3.37	0.00	0.00

Staphylococcus	3.69	2.24	0.69	3.25	0.00	0.01
Scardovia	3.43	-3.61	0.76	-4.74	0.00	0.00
Prevotella_9	2.68	3.40	0.99	3.45	0.00	0.00
Butyrivibrio_2	1.13	-3.35	0.98	-3.41	0.00	0.00
Shuttleworthia	1.12	-2.32	0.76	-3.05	0.00	0.01
Solobacterium	0.90	-2.08	0.65	-3.22	0.00	0.01

ASV Differentially Abundant Species.

Methods: For the identification of the potential species, we used the "unoise3" command implemented in the usearch instead of using the "-cluster_otus" command to obtain the taxonomy data with species level prediction. The underlying dataset SILVA for OTUs classification has species level identification where application for OTUs. Only 27% of the SILVA dataset used has species level identification with many incomplete identifications. The species level prediction using a short tag like V4 region is debatable and thus used with caution. Thus, the species level analysis is presented in the supplementary data for readers as a valuable resource for the future development. We performed the same filtering criteria to filter the OTUs. The differentially abundant species as determined by DESeq2 using the ASV files calculated using the phyloseq package. Comparisons of the samples from stool and oral microbiomes for each of the four locations (Barbodos, Chile, South Africa, and Thailand) versus all other samples are included. In each table, only species with baseMean more than 50 are shown. The species identification column displays the genus name followed by the species name as many species have either unclassified or uncultured name tags.

Differentially abundant species using stool samples: -

Table S8. The differentially abundant species in Thailand stool samples obtained using DESeq2 algorithm with FDR < 0.05.

Species identification	padj	baseMean	log2FoldChange
Bacteroides Bacteroides_plebeius	0.00	585.44	2.97
Bacteroides Bacteroides_massiliensis	0.00	507.37	1.96
Alistipes Alistipes_unclassified	0.01	354.86	0.86
Bacteroides Bacteroides_coprocola_DSM_17136	0.00	320.19	3.35
Pseudobutyrivibrio uncultured_organism	0.03	263.73	-0.73
Prevotella_9 uncultured_bacterium	0.04	235.53	-1.50
Parabacteroides Parabacteroides_merdae	0.04	220.73	0.60
Bacteroides Bacteroides_caccae	0.10	199.18	0.55
Phascolarctobacterium uncultured_organism	0.07	190.37	0.98
Blautia Ruminococcus_sp._5_1_39BFAA	0.02	154.31	0.43
Ruminococcaceae_UCG-002 uncultured_bacterium	0.00	150.46	-1.55
Fusobacterium Fusobacterium_mortiferum	0.00	143.96	4.96
Escherichia-Shigella Escherichia_coli	0.00	143.95	1.44
Akkermansia Akkermansia_unclassified	0.00	142.16	-1.89
Prevotella_7 uncultured_organism	0.00	126.47	-13.25
Bacteroides Bacteroides_ovatus	0.00	125.40	-1.26

Sutterella Sutterella_unclassified	0.02	118.50	1.59
Ruminococcus_2 Ruminococcus_2_unclassified	0.01	111.26	-1.27
Subdoligranulum Subdoligranulum_unclassified	0.00	109.72	-1.38
Ruminococcaceae_UCG-014 Ruminococcaceae_UCG-014_unclassified	0.00	105.34	-3.04
Blautia Blautia_unclassified	0.00	104.17	-0.61
Ruminococcus_1 Ruminococcus_bicirculans	0.00	104.01	-1.89
Lachnospiraceae_UCG-004 uncultured_organism	0.08	96.17	0.49
Roseburia Roseburia_unclassified	0.05	91.54	0.62
Bacteroides uncultured_organism	0.00	76.18	-0.89
Faecalibacterium Faecalibacterium_unclassified	0.00	65.93	-0.79
Christensenellaceae_R-7_group uncultured_Christensenellaceae_bacterium	0.01	65.77	-2.04
Dorea uncultured_bacterium	0.02	56.73	-0.53
Megamonas uncultured_organism	0.00	54.98	3.37
Ruminococcaceae_UCG-005 Ruminococcaceae_UCG-005_unclassified	0.00	54.93	-1.20

Table S9. The differentially abundant species in Barbados stool samples obtained using DESeq2 algorithm with FDR < 0.05.

Species identification	padj	baseMean	log2FoldChange
Prevotella_9 uncultured_organism	0.00	6216.68	-3.53
Bacteroides Bacteroides_plebeius	0.00	570.96	-4.25
Alistipes Alistipes_unclassified	0.04	354.86	-0.88
Bacteroides Bacteroides_coprocola_DSM_17136	0.00	314.22	-4.76
Pseudobutyrivibrio uncultured_organism	0.01	263.73	1.04
Bacteroides Bacteroides_ovatus	0.00	138.73	1.38
Escherichia-Shigella Escherichia_coli	0.00	136.86	-1.86
Akkermansia Akkermansia_unclassified	0.00	128.95	-2.38
[Eubacterium]_coprostanoligenes_group human_gut_metagenome	0.01	108.57	-3.05
Lachnospiraceae_UCG-004 uncultured_organism	0.04	96.17	0.74
Lachnospiraceae_UCG-005 uncultured_organism	0.04	77.12	1.11
Bacteroides uncultured_organism	0.01	76.18	0.97
Fusobacterium Fusobacterium_mortiferum	0.00	64.29	-10.14
Ruminococcaceae_UCG-005 Ruminococcaceae_UCG-005_unclassified	0.00	52.57	-1.58

Table S10. The differentially abundant species in Chile stool samples obtained using DESeq2 algorithm with FDR < 0.05.

Species identification	padj	baseMean	log2FoldChange
Bacteroides Bacteroides_stercoris_ATCC_43183	0.01	640.65	-1.36
Bacteroides Bacteroides_plebeius	0.04	545.77	-2.21

Bacteroides Bacteroides_massiliensis	0.01	507.37	-1.69
Faecalibacterium uncultured_organism	0.00	493.64	-0.86
Bacteroides Bacteroides_coprocola_DSM_17136	0.01	298.92	-2.53
Prevotella_9 uncultured_bacterium	0.00	226.27	-3.64
Bifidobacterium Bifidobacterium_unclassified	0.00	203.09	-2.09
Fusobacterium Fusobacterium_mortiferum	0.00	139.63	-7.01
Akkermansia Akkermansia_unclassified	0.09	134.17	1.32
Escherichia-Shigella Escherichia_coli	0.00	132.41	-1.67
Fusicatenibacter Fusicatenibacter_unclassified	0.09	111.49	0.51
Blautia Blautia_unclassified	0.05	104.17	0.41
Lachnospiraceae_UCG-004 uncultured_organism	0.01	96.17	-0.80
Roseburia Roseburia_unclassified	0.04	80.90	-0.69
Lachnospiraceae_UCG-005 uncultured_organism	0.03	77.12	-1.01
Faecalibacterium Faecalibacterium_unclassified	0.10	65.93	-0.42
Ruminococcaceae_UCG-005 Ruminococcaceae_UCG-005_unclassified	0.01	54.93	1.05

Table S11. The differentially abundant species in South Africa stool samples obtained using DESeq2 algorithm with FDR < 0.05.

**Species identification**	**padj**	**baseMean**	**log2FoldChange**
Bacteroides Bacteroides_unclassified	0.04	3785.98	-0.64
Bacteroides Bacteroides_plebeius	0.00	580.02	-5.90
Faecalibacterium uncultured_bacterium	0.03	540.09	0.87
Faecalibacterium uncultured_organism	0.00	493.64	1.27
Alistipes Alistipes_unclassified	0.00	354.86	-1.70
Bacteroides Bacteroides_coprocola_DSM_17136	0.00	320.19	-11.96
Bifidobacterium Bifidobacterium_unclassified	0.00	203.09	2.06
Blautia Ruminococcus_sp._5_1_39BFAA	0.10	154.31	-0.47
Ruminococcaceae_UCG-002 uncultured_bacterium	0.00	150.46	1.69
Bacteroides Bacteroides_ovatus	0.00	136.56	-2.26
Fusicatenibacter Fusicatenibacter_unclassified	0.04	111.49	-0.83
Ruminococcus_2 Ruminococcus_2_unclassified	0.08	111.26	1.44
Subdoligranulum Subdoligranulum_unclassified	0.05	109.72	1.23
Blautia Blautia_unclassified	0.00	97.53	-0.77
Lachnospiraceae_UCG-004 uncultured_organism	0.01	96.17	-1.02
Faecalibacterium Faecalibacterium_unclassified	0.00	65.93	1.10
Fusobacterium Fusobacterium_mortiferum	0.04	53.55	-3.48
Parabacteroides Parabacteroides_unclassified	0.04	51.21	-0.68
Odoribacter Odoribacter_splanchnicus_DSM_20712	0.06	50.68	-0.82

Differentially abundant species using stool samples: -

Table S12. The differentially abundant species in Thailand oral samples obtained using DESeq2 algorithm with FDR < 0.05.

Species identification	padj	baseMean	log2FoldChange
Gemella uncultured_bacterium	0.00	5438.36	1.96
Veillonella Veillonella_unclassified	0.00	1443.92	-2.20
Neisseria uncultured_bacterium	0.00	1117.14	2.31
Prevotella_7 Prevotella_melaninogenica	0.00	984.78	-1.91
Granulicatella uncultured_bacterium	0.00	518.88	-3.94
Haemophilus Haemophilus_unclassified	0.00	430.20	4.25
Prevotella Prevotella_unclassified	0.00	392.71	-2.46
Prevotella_7 Prevotella_7_unclassified	0.00	306.71	-3.47
Streptococcus Streptococcus_salivarius_subsp._thermophilus	0.00	198.77	-1.88
Aggregatibacter uncultured_bacterium	0.00	189.23	1.89
Veillonella Veillonella_sp._oral_taxon_780	0.00	141.84	-3.27
Bergeyella uncultured_bacterium	0.00	140.49	1.76
Alloprevotella uncultured_Bacteroidetes_bacterium	0.00	120.11	-1.88
Neisseria Neisseria_oralis	0.05	115.84	2.18
Rothia uncultured_organism	0.01	79.67	-1.66
Prevotella Prevotella_nigrescens	0.06	72.41	-0.95
Prevotella Prevotella_pallens	0.00	67.17	-3.80
Prevotella Prevotella_oris	0.00	65.86	-1.40
Alloprevotella Alloprevotella_unclassified	0.00	65.68	-2.85
Selenomonas_3 Selenomonas_3_unclassified	0.07	60.16	-0.99
Streptococcus Streptococcus_sanguinis	0.00	57.55	1.41
Prevotella_6 Prevotella_6_unclassified	0.00	57.18	-3.30

Table S13. The differentially abundant species in Barbados oral samples obtained using DESeq2 algorithm with FDR < 0.05.

Species identification	padj	baseMean	log2FoldChange
Dialister uncultured_bacterium	0.00	5074.40	-3.83
Prevotella_9 uncultured_organism	0.00	465.67	-7.28
Cronobacter Cronobacter_muytjensii	0.04	295.20	-1.15
Actinomyces Actinomyces_sp._oral_clone_DR002	0.02	277.23	1.15
Stomatobaculum uncultured_bacterium	0.06	256.06	-1.98
Bacteroides Bacteroides_plebeius	0.00	229.52	-7.92
Succinivibrio Succinivibrio_unclassified	0.00	123.16	-2.13
Alistipes Alistipes_sp._627	0.02	108.03	1.36
Actinomyces Actinomyces_unclassified	0.01	87.23	-2.23
Atopobium Atopobium_unclassified	0.00	63.43	-1.78
Actinomyces Actinomyces_odontolyticus	0.00	62.11	-3.21
Bacteroides Bacteroides_coprocola_DSM_17136	0.00	54.64	-10.36

Table S14. The differentially abundant species in Chile oral samples obtained using DESeq2 algorithm with FDR < 0.05.

Species identification	padj	baseMean	log2FoldChange
Streptococcus Streptococcus_unclassified	0.04	5639.96	1.21
Veillonella Veillonella_unclassified	0.00	1443.92	-3.42
Prevotella_7 Prevotella_melaninogenica	0.00	984.78	-3.05
Granulicatella uncultured_bacterium	0.07	518.88	-1.99
Prevotella Prevotella_unclassified	0.00	392.71	-2.52
Prevotella_7 Prevotella_7_unclassified	0.04	306.71	-1.72
Streptococcus Streptococcus_salivarius_subsp._thermophilus	0.01	198.77	-1.97
Actinomyces Actinomyces_unclassified	0.01	144.92	-1.82
Leptotrichia Leptotrichia_unclassified	0.08	138.49	-1.00
Alloprevotella uncultured_Bacteroidetes_bacterium	0.04	120.11	-1.73
Veillonella Veillonella_sp._oral_taxon_780	0.08	95.01	-2.17
Neisseria Neisseria_oralis	0.01	67.12	-3.43
Prevotella Prevotella_pallens	0.00	66.71	-4.45
Alloprevotella Alloprevotella_unclassified	0.07	65.68	-1.65
Prevotella_6 Prevotella_6_unclassified	0.08	57.18	-1.76

Table S15. The differentially abundant species in South Africa oral samples obtained using DESeq2 algorithm with FDR < 0.05.

Species identification	padj	baseMean	log2FoldChange
Gemella uncultured_bacterium	0.02	5438.36	-1.52
Veillonella Veillonella_unclassified	0.00	1443.92	1.87
Neisseria uncultured_bacterium	0.00	1117.14	-1.97
Prevotella_7 Prevotella_melaninogenica	0.00	984.78	2.42
Granulicatella uncultured_bacterium	0.00	518.88	2.44
Prevotella Prevotella_unclassified	0.00	392.71	2.64
Prevotella_7 Prevotella_7_unclassified	0.08	306.71	1.27
Rothia Rothia_unclassified	0.00	247.89	-2.06
Porphyromonas uncultured_bacterium	0.04	237.09	1.21
Veillonella Veillonella_sp._oral_taxon_780	0.00	218.16	4.18
Streptococcus Streptococcus_salivarius_subsp._thermophilus	0.00	198.77	1.70
Aggregatibacter uncultured_bacterium	0.02	189.23	-1.41
Alloprevotella uncultured_Bacteroidetes_bacterium	0.00	156.69	2.16
Lautropia uncultured_bacterium	0.00	153.84	-2.55
Bergeyella uncultured_bacterium	0.03	140.49	-1.17
Haemophilus Haemophilus_unclassified	0.00	121.16	-1.86
Actinomyces Actinomyces_unclassified	0.06	116.57	-1.08
Alloprevotella Alloprevotella_unclassified	0.01	65.68	1.89
Prevotella_6 Prevotella_6_unclassified	0.06	57.18	1.60

Table S16. SRA IDs of the samples used in the study for analysis.

NCBI SRR ID	Sample ID	NCBI Experiment Name
SRR9606770	Q0000214G	SRX6370643
SRR9606771	Q0000215G	SRX6370642
SRR9606772	Q0000207G	SRX6370641
SRR9606773	Q0000208G	SRX6370640
SRR9606774	Q0000209G	SRX6370639
SRR9606775	Q0000210G	SRX6370638
SRR9606776	Q0000216G	SRX6370637
SRR9606777	Q0000217G	SRX6370636
SRR9606778	Q0000124O	SRX6370635
SRR9606780	Q0000113O	SRX6370633
SRR9606781	Q0000217O	SRX6370632
SRR9606785	Q0000223O	SRX6370628
SRR9606787	Q0000225O	SRX6370626
SRR9606788	Q0000226O	SRX6370625
SRR9606790	Q0000228O	SRX6370623
SRR9606791	Q0000017O	SRX6370622
SRR9606792	Q0000016O	SRX6370621
SRR9606793	Q0000029O	SRX6370620
SRR9606794	Q0000028O	SRX6370619
SRR9606795	Q0000023O	SRX6370618
SRR9606796	Q0000022O	SRX6370617
SRR9606798	Q0000134G	SRX6370615
SRR9606799	Q0000126G	SRX6370614
SRR9606800	Q0000132G	SRX6370613
SRR9606803	Q0000135G	SRX6370610
SRR9606804	Q0000136G	SRX6370609
SRR9606805	Q0000140G	SRX6370608
SRR9606806	Q0000142G	SRX6370607
SRR9606807	Q0000167G	SRX6370606
SRR9606808	Q0000168G	SRX6370605
SRR9606809	Q0000154G	SRX6370604
SRR9606810	Q0000155G	SRX6370603
SRR9606811	Q0000156G	SRX6370602
SRR9606812	Q0000159G	SRX6370601
SRR9606814	Q0000163G	SRX6370599
SRR9606819	Q0000029G	SRX6370594
SRR9606820	Q0000028G	SRX6370593
SRR9606821	Q0000023G	SRX6370592
SRR9606822	Q0000022G	SRX6370591

SRR9606823	Q0000021G	SRX6370590
SRR9606824	Q0000020G	SRX6370589
SRR9606828	Q0000396G	SRX6370585
SRR9606829	Q0000255G	SRX6370584
SRR9606830	Q0000258G	SRX6370583
SRR9606831	Q0000259G	SRX6370582
SRR9606832	Q0000260G	SRX6370581
SRR9606835	Q0000393G	SRX6370578
SRR9606836	Q0000394G	SRX6370577
SRR9606837	Q0000583O	SRX6370576
SRR9606838	GU000560G	SRX6370575
SRR9606839	Q0000552G	SRX6370574
SRR9606840	OR000550G	SRX6370573
SRR9606841	OR000549G	SRX6370572
SRR9606842	OR000547G	SRX6370571
SRR9606843	Q0000546G	SRX6370570
SRR9606845	Q0000544G	SRX6370568
SRR9606846	OR000563G	SRX6370567
SRR9606847	Q0000562G	SRX6370566
SRR9606848	Q0000400O	SRX6370565
SRR9606849	Q0000401O	SRX6370564
SRR9606850	Q0000402O	SRX6370563
SRR9606852	Q0000396O	SRX6370561
SRR9606854	Q0000398O	SRX6370559
SRR9606855	Q0000399O	SRX6370558
SRR9606856	Q0000121O	SRX6370557
SRR9606857	Q0000119O	SRX6370556
SRR9606858	Q0000118O	SRX6370555
SRR9606860	Q0000404O	SRX6370553
SRR9606861	Q0000111O	SRX6370552
SRR9606864	GU000566O	SRX6370549
SRR9606866	Q0000564O	SRX6370547
SRR9606868	Q0000562O	SRX6370545
SRR9606869	OR000563O	SRX6370544
SRR9606871	GU000560O	SRX6370542
SRR9606872	Q0000405O	SRX6370541
SRR9606873	Q0000430O	SRX6370540
SRR9606874	Q0000431O	SRX6370539
SRR9606875	Q0000432O	SRX6370538
SRR9606876	Q0000190O	SRX6370537
SRR9606877	Q0000189O	SRX6370536

SRR9606878	Q0000433O	SRX6370535
SRR9606884	Q0000187O	SRX6370529
SRR9606885	Q0000186O	SRX6370528
SRR9606887	Q0000427O	SRX6370526
SRR9606888	Q0000428O	SRX6370525
SRR9606889	Q0000429O	SRX6370524
SRR9606890	OR000531O	SRX6370523
SRR9606891	Q0000125G	SRX6370522
SRR9606892	Q0000124G	SRX6370521
SRR9606894	Q0000116G	SRX6370519
SRR9606895	Q0000113G	SRX6370518
SRR9606896	Q0000111G	SRX6370517
SRR9606898	Q0000121G	SRX6370515
SRR9606899	Q0000119G	SRX6370514
SRR9606900	Q0000118G	SRX6370513
SRR9606901	GU000618O	SRX6370512
SRR9606902	OR000619O	SRX6370511
SRR9606904	OR000584O	SRX6370509
SRR9606905	Q0000620O	SRX6370508
SRR9606906	Q0000640G	SRX6370507
SRR9606907	OR000641G	SRX6370506
SRR9606908	OR000631G	SRX6370505
SRR9606910	GU000628G	SRX6370503
SRR9606911	Q0000630G	SRX6370502
SRR9606912	Q0000637G	SRX6370501
SRR9606913	OR000639G	SRX6370500
SRR9606914	Q0000635G	SRX6370499
SRR9606915	GU000636G	SRX6370498
SRR9606916	Q0000223G	SRX6370497
SRR9606917	Q0000221G	SRX6370496
SRR9606918	Q0000220G	SRX6370495
SRR9606921	Q0000226G	SRX6370492
SRR9606922	Q0000225G	SRX6370491
SRR9606924	Q0000229G	SRX6370489
SRR9606925	Q0000228G	SRX6370488
SRR9606926	Q0000220O	SRX6370487
SRR9606927	Q0000221O	SRX6370486
SRR9606928	OR000543G	SRX6370485
SRR9606931	Q0000537G	SRX6370482
SRR9606932	OR000539G	SRX6370481
SRR9606934	Q0000535G	SRX6370479

SRR9606935	OR000532G	SRX6370478
SRR9606936	GU000533G	SRX6370477
SRR9606938	Q0000241O	SRX6370475
SRR9606939	Q0000240O	SRX6370474
SRR9606940	Q0000239O	SRX6370473
SRR9606941	Q0000237O	SRX6370472
SRR9606942	Q0000236O	SRX6370471
SRR9606943	Q0000234O	SRX6370470
SRR9606944	Q0000233O	SRX6370469
SRR9606945	Q0000231O	SRX6370468
SRR9606946	Q0000229O	SRX6370467
SRR9606947	Q0000008O	SRX6370466
SRR9606949	Q0000004O	SRX6370464
SRR9606950	Q0000005O	SRX6370463
SRR9606951	Q0000001O	SRX6370462
SRR9606953	Q0000438G	SRX6370460
SRR9606954	Q0000439G	SRX6370459
SRR9606955	Q0000020O	SRX6370458
SRR9606958	Q0000413G	SRX6370455
SRR9606960	Q0000411G	SRX6370453
SRR9606961	Q0000412G	SRX6370452
SRR9606962	Q0000409G	SRX6370451
SRR9606963	Q0000410G	SRX6370450
SRR9606965	Q0000408G	SRX6370448
SRR9606966	Q0000415G	SRX6370447
SRR9606968	OR000606O	SRX6370445
SRR9606970	Q0000605O	SRX6370443
SRR9606971	Q0000598O	SRX6370442
SRR9606973	OR000604O	SRX6370440
SRR9606974	Q0000635O	SRX6370439
SRR9606976	Q0000637O	SRX6370437
SRR9606977	GU000636O	SRX6370436
SRR9606978	Q0000640O	SRX6370435
SRR9606979	OR000639O	SRX6370434
SRR9606980	Q0000642O	SRX6370433
SRR9606981	OR000641O	SRX6370432
SRR9606983	OR000643O	SRX6370430
SRR9606984	Q0000404G	SRX6370429
SRR9606985	Q0000403G	SRX6370428
SRR9606986	Q0000402G	SRX6370427
SRR9606987	Q0000401G	SRX6370426

SRR9606988	Q0000400G	SRX6370425
SRR9606989	Q0000399G	SRX6370424
SRR9606990	Q0000398G	SRX6370423
SRR9606992	Q0000406G	SRX6370421
SRR9606993	Q0000405G	SRX6370420
SRR9606994	OR000592G	SRX6370419
SRR9606996	Q0000590G	SRX6370417
SRR9607000	OR000595G	SRX6370413
SRR9607002	OR000601G	SRX6370411
SRR9607003	Q0000602G	SRX6370410
SRR9607004	Q0000409O	SRX6370409
SRR9607005	Q0000408O	SRX6370408
SRR9607006	Q0000407O	SRX6370407
SRR9607007	Q0000406O	SRX6370406
SRR9607008	Q0000413O	SRX6370405
SRR9607009	Q0000412O	SRX6370404
SRR9607010	Q0000411O	SRX6370403
SRR9607011	Q0000410O	SRX6370402
SRR9607014	Q0000084O	SRX6370399
SRR9607017	Q0000099O	SRX6370396
SRR9607019	Q0000095O	SRX6370394
SRR9607020	OR000577O	SRX6370393
SRR9607021	OR000576O	SRX6370392
SRR9607022	Q0000579O	SRX6370391
SRR9607023	OR000578O	SRX6370390
SRR9607025	OR000627G	SRX6370388
SRR9607027	GU000574O	SRX6370386
SRR9607029	Q0000623G	SRX6370384
SRR9607030	Q0000624G	SRX6370383
SRR9607031	OR000625G	SRX6370382
SRR9607032	Q0000617G	SRX6370381
SRR9607033	GU000618G	SRX6370380
SRR9607034	OR000619G	SRX6370379
SRR9607038	Q0000617O	SRX6370375
SRR9607039	Q0000620G	SRX6370374
SRR9607040	OR000643G	SRX6370373
SRR9607041	Q0000642G	SRX6370372
SRR9607045	Q0000202O	SRX6370368
SRR9607046	Q0000008G	SRX6370367
SRR9607048	Q0000201O	SRX6370365
SRR9607049	Q0000197O	SRX6370364

SRR9607050	Q0000199O	SRX6370363
SRR9607051	Q0000192O	SRX6370362
SRR9607053	GU000522O	SRX6370360
SRR9607054	Q0000017G	SRX6370359
SRR9607055	Q0000016G	SRX6370358
SRR9607056	Q0000204O	SRX6370357
SRR9607057	Q0000205O	SRX6370356
SRR9607060	Q0000099G	SRX6370353
SRR9607066	Q0000095G	SRX6370347
SRR9607071	Q0000190G	SRX6370342
SRR9607072	Q0000192G	SRX6370341
SRR9607074	Q0000189G	SRX6370339
SRR9607075	Q0000186G	SRX6370338
SRR9607076	Q0000187G	SRX6370337
SRR9607082	GU000612O	SRX6370331
SRR9607083	OR000607O	SRX6370330
SRR9607084	Q0000609O	SRX6370329
SRR9607085	OR000531G	SRX6370328
SRR9607086	OR000530G	SRX6370327
SRR9607090	GU000522G	SRX6370323
SRR9607091	OR000528G	SRX6370322
SRR9607093	Q0000437O	SRX6370320
SRR9607095	Q0000439O	SRX6370318
SRR9607097	Q0000252O	SRX6370316
SRR9607099	Q0000245O	SRX6370314
SRR9607100	Q0000247O	SRX6370313
SRR9607102	Q0000244O	SRX6370311
SRR9607103	Q0000250O	SRX6370924
SRR9607107	Q0000432G	SRX6370920
SRR9607108	Q0000431G	SRX6370919
SRR9607110	Q0000433G	SRX6370917
SRR9607111	Q0000428G	SRX6370916
SRR9607112	Q0000427G	SRX6370915
SRR9607113	Q0000430G	SRX6370914
SRR9607115	OR000586O	SRX6370912
SRR9607117	Q0000590O	SRX6370910
SRR9607119	Q0000437G	SRX6370908
SRR9607121	Q0000021O	SRX6370906
SRR9607122	GU000585O	SRX6370905
SRR9607123	Q0000422G	SRX6370904
SRR9607124	Q0000421G	SRX6370903

SRR9607125	Q0000424G	SRX6370902
SRR9607126	Q0000423G	SRX6370901
SRR9607129	Q0000420G	SRX6370898
SRR9607131	Q0000426G	SRX6370896
SRR9607132	Q0000425G	SRX6370895
SRR9607134	Q0000167O	SRX6370893
SRR9607135	Q0000163O	SRX6370892
SRR9607137	Q0000159O	SRX6370890
SRR9607139	Q0000207O	SRX6370888
SRR9607140	Q0000156O	SRX6370887
SRR9607142	Q0000154O	SRX6370885
SRR9607143	Q0000206O	SRX6370884
SRR9607144	Q0000244G	SRX6370883
SRR9607145	Q0000216O	SRX6370882
SRR9607148	OR000539O	SRX6370879
SRR9607150	Q0000084G	SRX6370877
SRR9607155	Q0000070G	SRX6370872
SRR9607159	OR000592O	SRX6370868
SRR9607160	Q0000234G	SRX6370867
SRR9607161	Q0000236G	SRX6370866
SRR9607162	Q0000231G	SRX6370865
SRR9607163	Q0000233G	SRX6370864
SRR9607164	Q0000240G	SRX6370863
SRR9607165	Q0000241G	SRX6370862
SRR9607166	Q0000237G	SRX6370861
SRR9607167	Q0000239G	SRX6370860
SRR9607174	OR000582O	SRX6370853
SRR9607175	Q0000579G	SRX6370852
SRR9607176	OR000578G	SRX6370851
SRR9607177	OR000581G	SRX6370850
SRR9607178	Q0000580G	SRX6370849
SRR9607179	Q0000583G	SRX6370848
SRR9607180	OR000582G	SRX6370847
SRR9607181	GU000585G	SRX6370846
SRR9607182	OR000584G	SRX6370845
SRR9607184	OR000586G	SRX6370843
SRR9607189	Q0000062O	SRX6370838
SRR9607190	Q0000070O	SRX6370837
SRR9607196	OR000528O	SRX6370831
SRR9607197	OR000530O	SRX6370830
SRR9607198	OR000615G	SRX6370829

SRR9607200	GU000533O	SRX6370827
SRR9607202	Q0000535O	SRX6370825
SRR9607203	Q0000537O	SRX6370824
SRR9607204	OR000607G	SRX6370823
SRR9607205	OR000606G	SRX6370822
SRR9607206	Q0000605G	SRX6370821
SRR9607207	OR000604G	SRX6370820
SRR9607209	GU000612G	SRX6370818
SRR9607211	Q0000609G	SRX6370816
SRR9607213	OR000572O	SRX6370814
SRR9607215	Q0000210O	SRX6370812
SRR9607216	Q0000214O	SRX6370811
SRR9607218	Q0000060O	SRX6370809
SRR9607220	Q0000209O	SRX6370807
SRR9607221	Q0000208O	SRX6370806
SRR9607222	Q0000056O	SRX6370805
SRR9607227	Q0000215O	SRX6370800
SRR9607231	OR000650O	SRX6370796
SRR9607232	OR000581O	SRX6370795
SRR9607234	Q0000580O	SRX6370793
SRR9607235	OR000532O	SRX6370792
SRR9607238	Q0000143G	SRX6370789
SRR9607239	Q0000147G	SRX6370788
SRR9607240	Q0000146G	SRX6370787
SRR9607242	Q0000148G	SRX6370785
SRR9607246	Q0000152G	SRX6370781
SRR9607247	Q0000155O	SRX6370780
SRR9607248	OR000650G	SRX6370779
SRR9607250	Q0000178G	SRX6370777
SRR9607252	Q0000176G	SRX6370775
SRR9607253	Q0000175G	SRX6370774
SRR9607255	Q0000173G	SRX6370772
SRR9607256	Q0000172G	SRX6370771
SRR9607259	Q0000415O	SRX6370768
SRR9607260	Q0000206G	SRX6370767
SRR9607261	Q0000205G	SRX6370766
SRR9607262	Q0000202G	SRX6370765
SRR9607263	Q0000201G	SRX6370764
SRR9607264	Q0000204G	SRX6370763
SRR9607266	Q0000197G	SRX6370761
SRR9607269	Q0000199G	SRX6370758

SRR9607272	OR000577G	SRX6370755
SRR9607273	Q0000624O	SRX6370754
SRR9607274	Q0000623O	SRX6370753
SRR9607276	OR000625O	SRX6370751
SRR9607277	GU000628O	SRX6370750
SRR9607278	OR000627O	SRX6370749
SRR9607279	Q0000564G	SRX6370748
SRR9607281	GU000566G	SRX6370746
SRR9607284	OR000572G	SRX6370743
SRR9607286	GU000574G	SRX6370741
SRR9607288	Q0000394O	SRX6370739
SRR9607290	Q0000255O	SRX6370737
SRR9607292	Q0000259O	SRX6370735
SRR9607293	Q0000258O	SRX6370734
SRR9607295	Q0000260O	SRX6370732
SRR9607296	Q0000393O	SRX6370731
SRR9607298	Q0000125O	SRX6370729
SRR9607299	Q0000126O	SRX6370728
SRR9607300	Q0000132O	SRX6370727
SRR9607302	Q0000134O	SRX6370725
SRR9607303	Q0000135O	SRX6370724
SRR9607304	Q0000136O	SRX6370723
SRR9607307	Q0000140O	SRX6370720
SRR9607309	OR000595O	SRX6370718
SRR9607310	Q0000423O	SRX6370717
SRR9607311	Q0000602O	SRX6370716
SRR9607312	OR000601O	SRX6370715
SRR9607314	Q0000422O	SRX6370713
SRR9607315	Q0000421O	SRX6370712
SRR9607317	Q0000152O	SRX6370710
SRR9607319	Q0000148O	SRX6370708
SRR9607320	Q0000147O	SRX6370707
SRR9607323	Q0000143O	SRX6370704
SRR9607324	Q0000142O	SRX6370703
SRR9607325	Q0000146O	SRX6370702
SRR9607327	OR000542G	SRX6370700
SRR9607328	OR000631O	SRX6370699
SRR9607330	Q0000178O	SRX6370697
SRR9607331	Q0000173O	SRX6370696
SRR9607333	Q0000175O	SRX6370694
SRR9607334	Q0000176O	SRX6370693

SRR9607335	Q0000168O	SRX6370692
SRR9607338	Q0000172O	SRX6370689
SRR9607342	Q0000630O	SRX6370685
SRR9607345	Q0000001G	SRX6370682
SRR9607346	Q0000005G	SRX6370681
SRR9607347	Q0000004G	SRX6370680
SRR9607349	Q0000062G	SRX6370678
SRR9607351	Q0000060G	SRX6370676
SRR9607355	Q0000056G	SRX6370672
SRR9607359	Q0000245G	SRX6370668
SRR9607362	Q0000247G	SRX6370665
SRR9607363	Q0000250G	SRX6370664
SRR9607365	Q0000252G	SRX6370662
SRR9607366	Q0000420O	SRX6370661
SRR9607368	Q0000253G	SRX6370659
SRR9607369	Q0000425O	SRX6370658
SRR9607370	Q0000424O	SRX6370657
SRR9607371	OR000615O	SRX6370656
SRR9607372	Q0000552O	SRX6370655
SRR9607373	OR000550O	SRX6370654
SRR9607374	OR000549O	SRX6370653
SRR9607375	OR000547O	SRX6370652
SRR9607376	Q0000546O	SRX6370651
SRR9607378	Q0000544O	SRX6370649
SRR9607379	OR000543O	SRX6370648
SRR9607380	OR000542O	SRX6370647
